# Religiosity and spirituality in the prevention and management of depression and anxiety in young people: a systematic review and meta-analysis

**DOI:** 10.1186/s12888-023-05091-2

**Published:** 2023-10-10

**Authors:** Shilpa Aggarwal, Judith Wright, Amy Morgan, George Patton, Nicola Reavley

**Affiliations:** 1 Public Health Foundation of India, Gurgaon, Haryana India; 2 Centre for Adolescent Health, Murdoch Children’s Research Institute, 50 Flemington Road, Melbourne, VIC 3052 Australia; 3 Deakin University, Geelong, Australia; 4 Centre for Mental Health, Melbourne School of Population and Global Health, University of Melbourne, Victoria, Australia; 5 Department of Paediatrics, University of Melbourne, Melbourne, VIC Australia

**Keywords:** Religious and spiritual beliefs, Depression, Anxiety, Prevention, Management, Young people

## Abstract

Historically, religion has had a central role in shaping the psychosocial and moral development of young people. While religiosity and spirituality have been linked to positive mental health outcomes in adults, their role during the developmental context of adolescence, and the mechanisms through which such beliefs might operate, is less well understood. Moreover, there is some evidence that negative aspects of religiosity are associated with poor mental health outcomes. Guided by lived experience consultants, we undertook a systematic review and quality appraisal of 45 longitudinal studies and 29 intervention studies identified from three electronic databases (Medline, PsycINFO and Scopus) exploring the role of religiosity and spiritual involvement (formal and informal) in prevention and management of depression and anxiety in young people aged 10 to 24 years. Most studies were from high-income countries and of low to moderate quality. Meta-analysis of high-quality longitudinal studies (assessed using Joanna Briggs Institute critical appraisal tools, *n* = 25) showed a trend towards association of negative religious coping (i.e., feeling abandoned by or blaming God) with greater depressive symptoms over time (Pearson’s *r* = 0.09, 95% confidence interval (CI) -0.009, 0.188) whereas spiritual wellbeing was protective against depression (Pearson’s *r* = -0.153, CI -0.187, -0.118). Personal importance of religion was not associated with depressive symptoms overall (Pearson’s *r* = -0.024, CI-0.053, 0.004). Interventions that involved religious and spiritual practices for depression and anxiety in young people were mostly effective, although the study quality was typically low and the heterogeneity in study designs did not allow for a meta-analysis. The lived experience consultants described spirituality and religious involvement as central to their way of life and greatly valued feeling watched over during difficult times. While we require more evidence from low- and middle-income countries, in younger adolescents and for anxiety disorders, the review provides insight into how spirituality and religious involvement could be harnessed to design novel psychological interventions for depression and anxiety in young people.

**Review Registration**

The systematic review was funded by Wellcome Trust Mental Health Priority Area 'Active Ingredients' 2021 commission and registered with PROSPERO 2021 (CRD42021281912).

**Supplementary Information:**

The online version contains supplementary material available at 10.1186/s12888-023-05091-2.

## Introduction

Religious and spiritual beliefs are complex multidimensional social phenomena that incorporate both subjective individual quests to find purpose and meaning in life and those associated with a specific religion [1, 2]. Aspects of such beliefs include: engagement in organisational and formal religious practices such as attending church services, religious youth groups; personal importance of religion (salience) i.e. importance of religious faith in shaping daily life including private religious/ spiritual activities such as prayer and meditation; spiritual wellbeing i.e. a sense of life-meaning, belonging and purpose; and religious coping including both positive (i.e. looking to God for strength, and support) and negative (i.e. reappraisals of God's powers, feeling abandoned by or blaming God) religious coping [3, 4].

Historically, religion has had a central role in shaping the psychosocial and moral development of young people [5]. For many, adolescence is a key period for exploration of religious and spiritual beliefs as they relate to purpose of life, vocation, and formation of relationships outside the home [6]. The relatively recent lengthening of adolescence due to earlier onset of puberty and delayed role transitions in the 20 s, also means a longer period during which young people can develop their religious and spiritual beliefs, potentially resulting in a deeper and more sophisticated understanding [7]. Young people develop a sense of identity during adolescence, with a dynamic exchange between ecological influences and personal agency. Religion can help foster a personal sense of hope, and meaning in life while increasing prosocial, community-oriented attitudes and behaviours during this developmental stage [8]. Furthermore, religious and spiritual beliefs can contribute to positive mental health through mechanisms such as religious morality, religious coping, and social connectedness due to shared beliefs [2, 9]. However, there is some evidence that those who experience religious or spiritual struggles, including anger with God, negative encounters with other members of their faith community or internal religious guilt or doubt, may be at higher risk of mental health problems [10–12].

In the context of a recent emphasis on social and cultural determinants of mental health in adolescence, the period in which mental health problems often have their first onset, questions have been raised about the extent to which religiosity and spirituality may be associated with anxiety and depression in young people [13–15]. Scientific studies spanning across various life stages have shown beneficial as well as unhelpful associations between religious and spiritual beliefs and mental health outcomes such as depression [16]. In a recent systematic review of 152 prospective studies, 49% reported at least one significant association between religiosity and spirituality and a better course of depression, although the strength of the associations was small (Cohen’s d =  − 0.18) [16].

However, the role of religious and spiritual beliefs in youth mental health is less well studied. A 2012 systematic review that examined the association between religious and spiritual beliefs in adolescents and emerging adults and a broad range of outcomes, such as wellbeing, depression, personality disorders and risk behaviours only included cross-sectional studies [17]. The review found an association of greater religiosity and spirituality with lower levels of depression symptoms but did not include longitudinal studies or cover the role of spiritual and religious beliefs in the management of psychological disorders [17]. Moreover, variability in measurement and operationalisation of religion and spirituality makes it difficult to compare and synthesise findings from longitudinal studies [18]. Although widely recognised as multidimensional, measurement of religious and spiritual beliefs is often limited to religious service attendance or religious affiliation, the mental health benefits of which could be due to social connectedness. There is a need to move beyond a focus on religious attendance alone and consider these beliefs as encompassing different dimensions. For intervention studies, large variations in study designs as well as limited pre- and post-assessment of religious and spiritual beliefs make it hard to identify consistent findings.

Thus, the aim of this review is to systematically review the evidence for the role of religious and spiritual involvement (formal and informal) in prevention and management of depression and anxiety in young people. A secondary aim is to explore the mechanisms through which these associations operate.

## Methods

### Systematic review

The systematic review was conducted as per PRISMA guidelines and registered with PROSPERO (CRD42021281912).

### Study eligibility criteria

Studies were considered eligible if they met the following criteria: (1) Prospective observational or intervention (including pre-post, quasi-experimental or randomized controlled trials) study design, (2) Assessed (a) the associations between religiosity and spirituality, and depression or anxiety; (b) the role of religiosity and spirituality in moderating the relationship between risk factors and depression or anxiety, and (c) the impact of interventions involving aspects of religiosity or spirituality on anxiety or depression, (3) Participant mean age between 10 to 24 years, (4) Used diagnostic criteria or validated scales (including sub-scales) to assess anxiety (e.g., GAD-7) or depression (e.g., CES-D), and (5) Journal articles published from 2000 onwards in English and peer-reviewed. Studies using measures of church attendance or religious service attendance as the only indicator of spirituality or religiosity were not considered eligible due to the focus on personal meaning and purpose rather than connection to a religious community alone.

### Identification and selection of studies

Three electronic databases (Medline, PsycINFO and Scopus) were searched for papers published in English between Jan 1, 2000 and July 21, 2021 to review the most recent and relevant evidence.

A search strategy was developed using a combination of medical subject headings (MeSH) terms and text words that covered religiosity or spirituality, depression or anxiety, and youth (See Appendix 1 for the full search strings). Obsessive–compulsive disorder (OCD) and post-traumatic stress disorder (PTSD) were included as part of the search due to their classification as anxiety disorders in previous editions of the Diagnostic and Statistical Manual of Mental Disorders (DSM- IV and earlier). Additional papers were identified from the reference lists of included studies and relevant reviews identified through the search and checked for eligibility.

Search results were imported into Covidence for title, abstract and full-text screening following de-duplication in Endnote. Title and abstract screening against the inclusion criteria was completed by one researcher (JW). Results that met inclusion criteria were full-text screened independently by two researchers (EC and JW) with disagreements resolved in consultation with a third reviewer (SA and NR).

### Data extraction and assessment of study quality

**Fig. 1:**
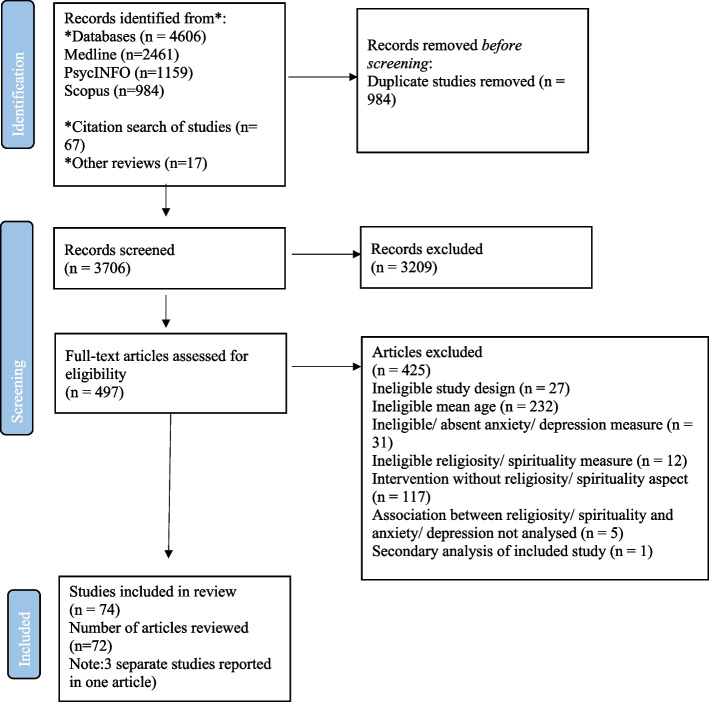
PRISMA flow diagram^75^

Data from each study were extracted by any two of the following researchers independently (SA, EC, NR and JW) into a data extraction template developed in Excel. Discrepancies were checked by a third researcher. Information extracted from studies included study aims and setting, population characteristics, data collection and analysis methods, findings, recommendations, strengths and limitations. The PRISMA diagram shows the final study numbers that were included in the review (See Fig. 1).

The Joanna Briggs Institute (JBI) critical appraisal tools were used to determine the risk of bias for included studies. The prospective observational studies were assessed using eleven questions (Yes/No/Unsure/Not applicable), the quasi-experimental studies were assessed using nine questions (Yes/No/Unsure/Not applicable), and the randomized controlled trial studies were assessed using 13 questions (Yes/No/Unsure/Not applicable). Each study was appraised by two independent reviewers (SA, NR or JW) for low, moderate or high risk of bias and any uncertainties or disagreements were resolved through discussion.

Scores for each study were totalled by summing the ‘Yes’ categories. Percentage scores were calculated for each study (with the denominator reflecting any NA categories). Studies with scores of 33.3% or lower were considered to be at high risk of bias (low quality), studies with scores between 33.4% and 66.7% were considered to be at moderate risk of bias (moderate quality) and studies with scores of 66.8% or above were considered to be at low risk of bias (high quality) (See Tables 1 and 2).

**Table 1:** Study characteristics of longitudinal studies

Author date	Country	Study name	Year of baseline in paper	Follow-up(s) after baseline	Overall study sample	Study sample size at baseline	Study setting	Sampling method	Gender (%F, %M)	Religion	Mean (SD) age, age range at baseline	Attrition (loss to each wave)	Mental Health Outcomes		Quality rating (risk of bias)
													**Anxiety**	**Depression**	
Ahles [19], 2016	Canada	NA	NR	T2-8: 7 consecutive weekly follow-ups	Undergraduate students from a small, private Christian university	320	Tertiary education	Convenience	71%F	NR	19.08 (2.1)	NR		CES-D SF	Low risk of bias
Ahmed [20], 2011^a^	USA	NR	NR	T2: 6—12 months	Homeless adolescents within a metropolitan area	186	Social welfare services	Probability	63%F	NR	16.01 (1.30), 13 to 17	NA		BSI	Low risk of bias
Benore [21], 2008	USA	NA	NR	T2: 1 month post hospitalization	Children and adolescents admitted for inpatient treatment of asthma	87	Hospital	Convenience	45%F, 55%M	Christian: 90% No affiliation: 10%	11.6 (2.45), 8 to 17	T2: 29%	STAIC	CES-D-SF	Moderate risk of bias
Berry & York [22], 2011	USA	NA	2008	T2-6: 5 monthly follow-ups	Religious public mid-western university students	214	Tertiary education	Convenience	70%F	Christian: 74% Islamic: 0.5% Jewish: 0.5% Other: 18% No affiliation: 15%	NR	T2: 5.1% T3: 7% T4: 6.5% T5: 10.7% T6: 16.8%		CES-D	Moderate risk of bias
Berry [23], 2012	USA	NA	2010 (Early in autumn term)	T2: Immediately following winter break T3: Immediately following spring break T4: Near the end of spring term	Incoming freshmen from colleges and universities	124	Tertiary education	Convenience	66%F	Christian: 29% Islamic: 26.6% Jewish: 20.2% No affiliation: 24.2%	Primarily 18 to 21 (95%)	NR		3 items from American College Health Association’s National College Health Assessment	Moderate risk of bias
Booth [24], 2008	USA	AddHealth	1995 (WI)	WII: 1996	Adolescents in grades 7 to 12	6507	School, community	Multistage, stratified, school-based, cluster	52%F	NR	15.7 (1.7)	WII: 26.5%		CES-D	Low risk of bias
Carpenter [25], 2012	USA	NR	NR	T2-T9: 8 weekly follow-ups	Adolescents in grades 9 to 12 from private religiously affiliated high schools	111	School	Convenience	72%F	Christian: 83.9% Jewish: 0.9% Hindu: 0.9% No affiliation: 14.3%	16.4 (1.33), 14.1 to 19.3	NR		CDI^@^	Moderate risk of bias
Chan [26], 2014	USA	NA	NA	T2: Two years T3: Four years	12^th^ grade young adults followed until four years out of public high school	744	School, community	Mixed: Convenience and random	55%F, 45%M	Christian: 44.1% Jewish: 5.1% Buddhist: 7.7% Other: 3.5% No affiliation: 39.6%	17.9 (0.38)	T2: 29% T3: 25%		CES-D	Low risk of bias
Chen [27], 2018	USA	Growing Up Today Study (GUTS)	1999	T2: 2007 T3: 2010 T4: 2013	Children (transitioning from adolescence to young adulthood) of participants in the Nurses Health Study	5,681 to 7,458	Community	Convenience	42.%M	NR	14.74 (1.66), 8 to 14	NR	Breslau’s 7-item short screening scale for PTSD	CES-D	Low risk of bias
Cotton [28], 2013	USA	NA	NR	T2: 11–14 months	Urban adolescents with asthma	151	Hospital	Convenience	60%F	Christian: 75% No preference: 23% Other: 2%	15.8 (1.8)	T2: 12.6%	MASC-10	CDI-S	Low risk of bias
Davis & Kiang [29], 2016	USA	NA	NA	WII-IV: 3 yearly follow ups	Asian American 9^th^ and 8^th^ grade public high school students	180	School	Stratified cluster	60%F	Christian: 33.8% Shamanism/animism: 25.4% Hinduism: 9.7% Jainism: .6% Islamic: 3.6% Atheism/agnostic: 1.8% Not specified: 15.8%	15.03 (0.92), 13 to 18	WII: 9% WIII: 13% WIV: 33%		CES-D, PANAS	Low risk of bias
Dew, Fuemmeler & Koenig [30], 2020^a^	USA	AddHealth	1995 (WI)	WII: 1996 WIII: 2001–2002 WIV: 2008–2009	Adolescents with parent interview data from WI	9416	School, community	Multistage, stratified, school-based, cluster	55%F, 45%M	NR	15.8 (1.6)	NA		CES-D	Low risk of bias
Dew, Kollins & Koenig [31], 2020^a^	USA	AddHealth	1995 (WI)	WII: 1996 WIII: 2001–2002 WIV: 2008–2009	8142 adolescents with religious data from all four waves and parent interview data from Wave I	8141	School, community	Multistage, stratified, school-based, cluster	55%F, 45%M	Christian: 83% Jewish: 0.7% Muslim: 0.2% Buddhist: 0.4% Hindu: 0.1% Other: 3% None: 11%	15.8 (1.6)	NA		CES-D	Moderate risk of bias
Dew [32], 2010	USA,	NA	NR	T2: 6 months	Adolescents from outpatient psychiatric clinics	145	Clinical	Convenience	42%F	Christian: 93% Other: 5% No affiliation: 5%	14.3 (1.8), 12 to 18	T2: 28%		CES-D	Low risk of bias
Goeke-Morey [33], 2014	Northern Ireland	NA	NR (WV)	WVI: 12 months	Youth from socially deprived wards in Belfast	667	Community	Stratified random	50%F, 50%M	Christian: 100%	15.75 (1.97)	NR	BSI	BSI, GHQ-12	Moderate risk of bias
Harker [34], 2001^a^	USA	AddHealth	1994–1995 (WI)	WII: 1996	Adolescents who participants in both WI and WII	13350	Community, school	Multistage, stratified, school-based, cluster	50%M	NR	15.04	NA		CES-D, BDI	Low risk of bias
Helms [35], 2015	USA	NA	NR	T2: 12 months	11^th^ grade students from rural, low-income high schools	313	School	Convenience	54%F	Christian: 80% Jewish: 0.3% Hindu: 0.6% Unsure/No affiliation: 14.7%	17.13 (0.48)	T2: 26%		MFQ	Low risk of bias
Horowitz & Garber [36], 2003	USA	NA	NR	T2-7: 6 yearly follow ups	6^th^ grade public school students	240	School	Judgmental	52.4%F	NR	11.86 (0.57)	NR		K- SADS-E, K-LIFE	Low risk of bias
Kasen [37], 2012	USA	NA	NR	T2: 10 years T3: 20 years	Offspring of depressed and non-depressed parents	263	Clinical, community	Judgmental	58.9%F	Christian: 79.5% Other affiliation: 20.5%	NR	T2: 15.6% T3: 29.4%		K- SADS-E, SADS-L,	Low risk of bias
Kent [38], 2020^a^	USA	AddHealth	1995 (WI)	WIII: 2001–2002 WIV: 2007–2008	Grades 7 to 12 adolescents with complete depressive symptoms measurement data	12248	School, community	Multistage, stratified, school-based cluster	54%F, 46%M	NR	NR	NA		CES-D	Moderate risk of bias
Kent & Bradshaw [39], 2020^a^	USA	AddHealth	1995 (WI)	WIII: 2001–2002 WIV: 2007–2008	Grades 7 to 12 adolescents with complete depressive symptom measurement data	12248	School, community	Multistage, stratified, school-based cluster	55%F	NR	13 to 18	NA		CES-D	Moderate risk of bias
Kim [40], 2002	South Korea	NA	NR	T2: 4 weeks	Undergraduate psychology students	113	Tertiary education	Convenience	35%F, 65%M	Christian: 44% Buddhist: 5% Other: 3% No preference: 48%	20 (Median age), 19 to 33	NR		PANAS	Low risk of bias
Lalayants [41], 2020	USA	National Survey of Child and Adolescent Wellbeing	2008–2009 (WI)	WII: 2009 – 2011 (18 months)	Youth who underwent Child Protective Services Investigation	5872	Social welfare services	Judgmental	41%F,59%M	NR	11 to 16	WII: 17% weighted attrition		CDI^@^	Moderate risk of bias
Le [42], 2007	USA	AddHealth	1994–1995 (WI)	WII: 1996	Grades 7 to 12 African American, Asian America, European American, Hispanic America or Native American adolescents	13317	School, community	Multistage, stratified, school-based cluster	NR	NR	NR	NR		CES-D	Moderate risk of bias
Liu [43], 2011	USA	NA	NR	WII: 6 months	7th grade adolescents of Mexican origin	189	School	Convenience	54%F	NR	12.29, 11 to 14	WII: 13.2%	YSR, CBCL	YSR^@^, CBCL	Low risk of bias
Malooly [44], 2017	USA	Adolescent Adjustment Project	2007	T2: 2008 T3: 2009	10th and 11th grade urban and rural public high school students	485	School	Convenience	54%F	NR	16.10 (0.67)	T2-3: 20%		CES-D	Low risk of bias
Miller [45], 2002	USA	NA	1977–1985	T2: 1992—1996	Youths with and without a history of childhood depression followed up into early adulthood	269	Clinical, community	Mixed: Convenience (with childhood depression) and random (without childhood depression)	47%F, 53%M	Christian: 68.4% Jewish: 10.7% Other: 12.6%	NR	NR		K-SADS-E, SADS-L	Moderate risk of bias
Paunesku [46], 2008	USA	AddHealth	1995 (WI)	WII: 1996 (12 months)	Adolescents in grades 7 to 12	6504	School, community	Multistage, stratified, school-based cluster	48%M	NR	16.1 (1.8)	WII: 26.5%		CES-D	Moderate risk of bias
Perez [47], 2009^a^	USA	NA	NR	T2: 6 months T3: 12 months	Public school students from 6^th^ through 9^th^ grades	1096	School	Convenience	50%F	NR	11 to 15	NA		CDI, 27-item modification of the BDI	Low risk of bias
Peterman [6], 2014^a^	USA	National Institute for Child Health and Human Development (NICHD) Study of Early Child Care	1991	T2: 3 years	Early adolescents followed up in mid-adolescence	952	School	Quota	48%F	NR	11 to 12	NA	YSR^@^	YSR^@^	Low risk of bias
Petts [48], 2008^a^	USA	AddHealth	1994–1995 (WI)	WII: 1996	Grade 7 to 12 adolescents who participated in both WI and WII, have information from parents and valid sample weights	13568	School, community	Multistage, stratified, school-based cluster	NR	NR	NR	NA		CES-D	Low risk of bias
Possel [49], 2011	USA	NA	NR	T2:4 months	High school students	273	School	Convenience	65%F	NR	15.29 (0.68)	NR		CDI ^@^	Moderate risk of bias
Ramos-Olazagasti [50], 2013	USA	Boricua Youth Study	2000	WII: NR WIII: 2004	Puerto Rican youth living in Standard Metropolitan Areas of San Juan and Caguas, Puerto Rico, and in the South Bronx, New York	1271	Community	Multistage probability	50%F, 50%M	NR	11.6	WII: 7.95% WIII: NR		NIMH-DISC-IV	Low risk of bias
Rasic [51], 2013	Canada	Adolescent Health Survey	2000–2001	T2: 2002–2003	10^th^ grade high school students	976	School	Convenience	51%F, 49%M	NR	15.7 (0.6)	T2: 38.1%		CES-D	Low risk of bias
Reynolds [52], 2014	USA	NA	2008–2009	T2: 2009–2012 (2 years)	Adolescents with cystic fibroisis or diabetes	128	Hospital	Convenience	53%M	Christian: 86% Other: 3% No affiliation: 11%	14.7 (1.8)	T2: 32%		Behavioral Assessment System for Children-Second Edition	Low risk of bias
Riley [53], 2016	USA	NA	NR	T2: 3 months	Sexual and gender minority (SGM), and heterosexual first-year urban Jesuit university students transitioning to and across college	2810	Tertiary education	Convenience	SGM: 56%F; heterosexual 71%F	NR	SGM: 18.38, Heterosexual: 18.49	T2: 36.8%	DASS-21	DASS-21	Moderate risk of bias
Sallquist [54], 2010	Indonesia	NR	NR	T2: 7.25 months T3: 1 year	7^th^ grade Muslim students	959	School	Convenience	53%F, 47%M	NR	13.33 (0.68), 11 to 16.92	NR	Kendall, Henin, Macdonald, and Treadwell's anxiety scale	CDI^#^	Moderate risk of bias
Smokowski [55], 2014^a^	USA	Rural Adaptation Project (RAP)	2011	T2: 2012 T3: 2013	Middle school students from rural, disadvantaged counties followed up into high school	4036	School	Mixed: Judgmental and random	52%F, 48%M	NR	12.8	NR	YSR^#^	YSR^#^	Moderate risk of bias
Smokowski [56], 2017^a^	USA	Rural Adaptation Project (RAP)	2011	T2: 2012 T3:2013 T4:2014	Middle school students from rural, disadvantaged counties followed up into high school	3715	School	Mixed: Judgmental and random	52%F, 48%M	NR	12.7 (1.05)	NR	YSR^#^	YSR^#^	Moderate risk of bias
Upenieks [57], 2021^a^	USA	National Study of Youth and Religion (NSYR)	2007–2008 (WIII)	WIV: 2013	Emerging adults	2432	Community	Random sample via telephone digit dialing	49% F	Christian: 84% Jewish: 5% Other/indeterminate: 11%	25.42, 20 to 32	NA		Modified 8-item version of CES-D	Moderate risk of bias
Van der Jagt-Jelsma [58], 2017	Netherlands	TRacking Adolescents’ Individual Lives Survey (TRAILS) clinical cohort	2004	T2-4: 3 follow-ups at 2 to 3 year intervals	Pre-adolescents with psychiatric problems referred to an outpatient psychiatry clinic followed up to young adulthood	543	Clinical	Judgmental	34% F, 66% M	NR	10 to 12	T2: 14.9% T3: 22.8% T4: 22.3%	ASEBA-YSR and ASR	ASEBA-YSR and ASR	Moderate risk of bias
Van Voorhees [59], 2008^a^	USA	AddHealth	1995 (WI)	WII: 1996	Adolescents in grades 7 to 12	6504	Community, school	Multistage, stratified, school-based cluster	NR	NR	NR	WII: 26.4%	Single question “Over the last twelve months, have you had trouble relaxing?”	CES-D	Low risk of bias
Wortman [60], 2012	USA	NA	NR	T2: 2 months	Undergraduate psychology students	140	Tertiary education	Convenience	64% F, 36% M	Christian: 100%	18.7 (0.98)	T2: 3%	Impact of event scale for PTSD	CES-D	Moderate risk of bias
Yang [61], 2017	Taiwan	NA	NR	T2: 6 months	High school students	2239	School	Multistage stratified cluster	47% F, 53% M	Buddhist or Daoist: 86.8%	16 to 18	T2: 12.9%		CES-D	Low risk of bias
Yeterian [62], 2015	USA,	NA	2006–2009	T2: 3 months T3: 6 months T4: 12 months (post-intake)	Adolescents who presented for treatment at an outpatient substance use disorder treatment facility	127	Clinical	Convenience	24%F, 76%M	NR	16.7 (1.2), 14 to 19	T2: 8.7% T3: 15.7% T4: 87.4%		BSI (global severity index)	Low risk of bias

ASEBA-YSR Achenbach System of Empirically Based Assessment- youth self-report, ASEBA-ASR- ASEBA- Adult self-report, BDI- Beck Depression Inventory, BSI- Brief Symptom Inventory, CBCL- Child Behavior Checklist [63], CES-D Center for Epidemiological Studies Depression Scale, CES-D SF- CES-D Short Form, CDI- Children’s depression inventory, CDI^@^- CDI [64], CDI^#^- CDI [65], CDI-S CDI, short version, GHQ-12- General Health Questionnaire, K-SADS-E- Schedule for Affective Disorders and Schizophrenia for School-Age Children-Epidemiologic version, K-LIFE- Longitudinal Interval Follow-up Evaluation for children, MASC-10, Multidimensional anxiety Scale for children-10 item, MFQ- Mood and Feelings Questionnaire PANAS-, NIMH-DISC-IV- National Institute of Mental Health Diagnostic Interview Schedule for Children IV, SADS-L-Schedule for Affective Disorders and Schizophrenia – Lifetime Version, STAIC-State-Trait Anxiety Inventory for Children, YSR^@^- Youth self-report [63], YSR^#^- Youth self-report (Achenbach & Rescorla, 2001), NA not available, NR not reported, F female, M male, T2,T3,T4- follow-up timepoints after T1 (considered as baseline), ^a^paper reported sub-sample of a larger study

**Table 2:** Study characteristics of intervention studies

Study name & year	Country	Study design	N (INT, CONT)	Follow-up points	Study population	%F	Mean age in years (SD), range	Description of intervention	Intervention length	Comparison condition & description	Mental health outcomes		Quality rating
											Anxiety	Depression	
Anastasi [66], 2008	USA	Quasi-experimental	30 (12,18)	post	College students	NR	NR	Recitation of the Rosary in a campus chapel	30 min	Watched 30 min video of religious content	STAI: ↓ (State), NS (Trait)	NA	Moderate risk of bias
Armento [67], 2012	USA	RCT	50 (25,25)	1-month	Undergraduate students with > 14 BDI-II score	62%F	20(2.75)	Single session of modified Behavioral Activation treatment and post-session activation completing religious activities	60 min + 2 week activation	Single session of supportive therapy	BAI: ↓ (Somatic) STAI: ↓ (Trait) (Maintained at follow-up)	BDI-II: ↓ (Maintained at follow-up)	Moderate risk of bias
Charkabi [68], 2014*	Iran	RCT	60 (30,30)	post	Secondary school students	NR	14.93(INT) 14.82(CONT)	Spiritual intelligence training to solve problems in everyday life	7-weekly sessions	No intervention	SCL-90-R: NS (INT only)	SCL-90-R: NS (INT only)	High risk of bias
Chen [27, 69], 2005	USA	RCT	177 (90,87)	1-month	College students	59%F	19.5(2.48)	Written exercise on an experience of trauma from a religious or spiritual perspective	3 writing sessions over one week	Written exercise on an experience of trauma	IES-R: NS (PTSD symptoms)	NA	High risk of bias
Chen [70], 2009	USA	RCT	Total = 215, Religious writing = 90 Conventional trauma writing = 87 CONT = 38	1-month	College students	57%F	19.3 (2.36)	Written exercise on an experience of trauma from a religious or spiritual perspective	3 writing sessions over one week	Written exercise on an experience of trauma (CTW) Written exercise on a trivial experience (CONT)	NA	CES-D: NS	High risk of bias
Chen [71], 2018	Taiwan	RCT	105 (52,53)	1-month	College students	54%F	20.7(3.47)	Written exercise on an experience of trauma from a religious or spiritual perspective	3 writing sessions over one week	Written exercise on an experience of trauma	IES-R: NS (PTSD symptoms)	NA	High risk of bias
Dami [72], 2019	Indonesia	Quasi-experimental	64 (32,32)	post, 5-weeks	Christian Religious Education Study Program students	69%F	18–30	Group counselling with a spiritual approach with sessions based on spiritual intelligence book and an emphasis on biblical principles	7 60-min sessions	NR	DASS-21 anxiety sub-scale: ↓	DASS-21 depression sub-scale: ↓	Moderate risk of bias
Ebrahimi [73], 2015	Iran	Quasi-experimental	40 (20,20)	Post	Male high school students	0%F	16.48(1.10)	Group cognitive-behavioural therapy with spiritual intelligence training component	8 sessions	NR	DASS-42 anxiety sub-scale: ↓	DASS-42 depression sub-scale: ↓	Moderate risk of bias
Hajra [74], 2021	Pakistan	Pre-post	60 Low religiosity: 30 High religiosity: 30	Post	University students with mild to moderate DASS-21 scores and low/high religiosity levels	61.7%F	18–30	Islamic art therapy involving completing patterns from an Islamic adult colouring book and free-hand calligraphy	14 daily 35-45min sessions	NA	DASS-21 anxiety sub-scale: ↓	DASS-21 depression sub-scale: ↓	Moderate risk of bias
Heidari [75], 2019	Iran	Quasi-experimental	60 (30,30)	Post	Individuals admitted to hospital for a suicide attempt	62%F	Predominantly emerging adults (80%)	Spiritual care counselling	8 sessions	NR	NA	BDI-II: ↓	High risk of bias
Kadafi [76], 2021	Indon esia	Quasi-experimental	14 (7,7)	Post	High school students with high anxiety	64%F	16–18	Islamic counselling sessions	3 40-min sessions	Individual counselling including Covid-19 information and advice	CAS: ↓	NA	Moderate risk of bias
Khaki [77], 2021	Iran	Quasi-experimental	152 (76,76)	Post	Female university students	100%F	NR	Religious and spiritual teachings	15 sessions	NR	GHQ-28 anxiety component: ↓	GHQ-28 depression component: ↓	High risk of bias
Khubalkar [78], 2009	India	Pre-post	12	Post	University graduate psychology students	50%F	21.58(NR), 21–23	Single integral meditation session	20 min	NA	STAI: ↓ (State), ↓ (Trait)	NA	Moderate risk of bias
Klawonn [79], 2019	USA	Quasi-experimental	21	Post	Graduate healthcare students with < = 16 on BDI	67%F	25(4), 23–29	Seminars on meditation with reading, breathing techniques, education on the kosha model, mindful movement/modified asana, and supine guided meditation	5 weekly 60-min seminars	5-week baseline control period	BAI: ↓	BDI: ↓	Moderate risk of bias
Lolla [80], 2018	India	Pre-post	52	Post	College students	NR	17–20	Listening to mantras at home	40-min at least four days per week over 60 days	NA	PGWBI anxiety dimension: ↓	PANAS negative affect subscale: ↓ PGWBI depressed mood dimension: NS	Moderate risk of bias
Maddix [81], 2018	USA	Quasi-experimental	60 (38,22)	Post	Undergraduate university students	NR	23(NR), 17–42	Spiritual disciplines taught and practiced in a history of spiritual practices and neuroscience course	7 weekly sessions	Not enrolled in course	STAI: NS (State and trait)	BDI: NS	Moderate risk of bias
Mastropieri [82], 2015	USA	Pre-post	13	Post	Emerging adult men from a homeless shelter transitional living program	0%F	22.38 (1.03), 20.27–23.38	Group psychotherapy with spiritual visualization	16 weekly 90-min sessions	NA	GAD-7: NS	PHQ-12: ↓	Low risk of bias
											GHQ-12: ↓ (Psychological distress)		
Pandya [83], 2021	India, Kenya, Nepal, South Africa	RCT	96 (48,48)	Post	Deaf and hard-of-hearing university students	37.5%F	INT: 21.48 (2.63) CONT: 21.34 (2.08)	Online spiritual counselling program	50 weekly 1-h sessions	Online relaxation sessions	GAD-7: ↓	NA	Low risk of bias
Penberthy [84], 2017	USA	Pre-post	205	Post	Undergraduate college students	68.1%F	20.7(1.6), 18–36	Buddhism Meditation and Modernity course covering knowledge and practice	13 bi-weekly lectures	NA	STAI: ↓	PANAS negative affect sub-scale: NS	Low risk of bias
Rickhi [85], 2015	Canada	RCT	62 (31,31)	Post, 16-weeks, 24-weeks	Adolescents and young adults with mild-to-moderate MDD	71%F	12–24	Online modules on spiritually informed principles (e.g., forgiveness, gratitude, compassion)	8 2-3h weekly modules	Waitlist	NA	CES-D (adolescents): ↓ HAMD (young adults): ↓	Low risk of bias
Safara [86], 2012	India, Iran	Quasi-experimental	64 (32,32, 32)	Post	Iranian female university students residing in Iran and India	100%F	18–45	Spiritual therapy	5 bi-weekly sessions	Cognitive therapy (CT) No intervention control (CONT)	NA	BDI: ↓ (INT vs CT; INT vs CONT)	Moderate risk of bias
Scott Richards [87], 2006	USA	RCT	122, (43, C: 35, ES: 44)	Post	Women receiving in-patient eating disorder treatment	100%F	21.2(6.6), 13–52	Read and participated in group discussion on *Spiritual Renewal: A Journey of Faith and Healing*, a self-help workbook on non-denominational spiritual readings and Judeo-Christian education materials	Reading + weekly 60-min group	Cognitive group: Read and participated in weekly group discussion on a CBT self-help workbook and attended Emotional support group: Weekly “open-topic” support group	OQ-45 anxiety subscale: ↓	OQ-45 depression subscale: ↓	High risk of bias
Singh 2021 [88], (Study 1)	India	Pre-post	42	Post, 21-days	Undergraduate college students	14.2%F	21.17(1.53), 19–27	Multi-component wellbeing program covering topics on mindfulness and meditation and videos of Indian spiritual leaders imparting psychological messages	14 25–40 min sessions	NA	DASS-21: ↓ (post), NS (follow-up)		Low risk of bias
Singh 2021 [88], (Study 2)	India	Pre-post	308	Post	Undergraduate college students	31.8%F	19.26(1.49), 18–26	Multi-component wellbeing program covering topics on mindfulness and meditation and videos of Indian spiritual leaders imparting psychological messages	14 25–40 min sessions	NA	DASS-21: NS		Low risk of bias
Singh 2021 [88], (Study 3)	India	Pre-post	112	Post	College freshman	28.6%F	18.24 (0.60), 18–20	Multi-component wellbeing program covering topics on mindfulness and meditation and videos of Indian spiritual leaders imparting psychological messages	NR	NA	DASS-21: NS		Moderate risk of bias
Smith [89], 2011*	USA	Quasi-experimental	81 (INT: 33, exercise yoga: 15, cont: 32)	Post	Undergraduate students with mild-to-moderate depression, anxiety and stress	50.5%F	21.15 (4.15)	Hatha yoga with a meditation based on one of the yamas or niyamas of yogic philosophy	Bi-weekly 60-min sessions for 7 weeks	Yoga-as-exercise: Hatha yoga only No intervention control	DASS anxiety sub-scale: ↓ (INT only)	DASS depression sub-scale: ↓(INT only)	Low risk of bias
Vazifeh Doust [90], 2020*	Iran	RCT	40 (20,20)	Post	Children undergoing hospital treatment	52.5%F	NR	Training program on spiritual care covering topics on trust, coping, prayer, thanksgiving, and patience	Five 45–60 min sessions over 4 weeks	Disease and care in chemotherapy pamphlets	MASC: ↓ (INT only)	NA	High risk of bias
Wachholtz [91], 2008	USA	RCT	92 (spiritual med: 25, Internal secular med: 22, External secular med: 23, Relaxation: 22)	Post	Meditation naïve psychology university students meeting criteria for vascular headache	90.4%F	19.1 (1.10)	Spiritual meditation involving aloud soft repetition of one of four spiritual meditation phrases (e.g., “God is peace”) to help with focus	20-min daily for one month	(1) Internal secular meditation, (2) External secular meditation, (3) Progressive muscle relaxation	STAI: ↓ (trait)	CES-D: NS PANAS negative affect sub-scale: ↓	Moderate risk of bias
Wachholtz [91], 2005	USA	RCT	84 (spiritual med: 25, Secular med: 21, Relaxation: 22)	2-weeks	University students	68%F	19.1 (1.03)	Spiritual meditation involving aloud soft repetition of one of four spiritual meditation phrases (e.g., “God is peace”) to help with focus	20-min daily for two weeks	(1) Secular meditation, (2) Relaxation	STAI: ↓	PANAS negative affect sub-scale: NS	High risk of bias

^*^ Quasi-experimental or RCT studies that reported within group differences only, *BAI* Beck Anxiety Inventory, *BDI* Beck Depression Inventory, *CAS* Coronavirus Anxiety Scale, *CES-D* Center for Epidemiologic Studies Depression Scale, *DASS* Depression Anxiety Stress Scales, *GAD-7* General Anxiety Disorder-7, *GHQ* General Health Questionnaire, *HAMD* Hamilton Depression Rating Scale (HAM-D), *IES-R* The Impact of Event Scale – Revised, *OQ-45* Outcome Questionnaire 45, *PANAS-N* Positive and Negative Affect Schedule, *PGWBI* Psychological General Wellbeing Index, *PHQ-12* Patient Health Questionnaire, *SCL-90-R* Symptom Checklist-90-Revised, *STAI* The State-Trait Anxiety Inventory, ↓ Significant reduction (*p* < .05) in anxiety or depressive symptoms, med- meditation, NS = no significant different in anxiety or depressive symptoms

### Data synthesis and analysis

Outcomes of high-quality longitudinal studies (*n* = 25) were pooled with Comprehensive Meta-Analysis (CMA) V2 software using a random-effects model. For all studies, effects were extracted (e.g., correlation coefficient, regression coefficient, odds ratios) with the accompanying measure of uncertainty (95% confidence interval (CI), standard error or p-value) or sample size and converted to correlation coefficients by the CMA software. Unstandardised regression coefficients were converted to standardised coefficients using the formula beta = b_x_ * (SD_x_/SD_y_) [92].

Measures of religiosity were categorised according to the following constructs: engagement in organisational and formal religious and spiritual practices; religious salience; religious coping (including both positive and negative religious coping); and spiritual wellbeing. The measurement tools used in each category are listed in Table 3. Measures of depression and anxiety are listed in Table 4.

**Table 3:** Measures of religiosity and spirituality

In many studies, aspects of religiosity such as faith or closeness to God, religious doubt, frequency of church attendance, prayer, and youth group participation, as well as the importance of religion were assessed by one or two questions with Likert scale options [24, 27, 29, 30, 34–38, 45, 50, 51, 54, 61, 93].
Measures of religiosity and spirituality largely fell into four categories: 1. Engagement in organisational and formal religious practices e.g., Religious Background and Behaviour Scale (RBB) [62, 94], and Organizational Religiousness Short Form [69, 95] 2. Personal importance of religion (religious salience) e.g., items assessing personal importance/confidence [96], Private Religious Practices Scale [97], Brief Multidimensional Measure of Religiousness/Spirituality (BMMRS) [98], Multidimensional Inventory of Black Identity ([29], Intrinsic Religious Motivation Scale [99], Religious Background and Behavior (RBB) scale [94], Religious Orientation Scale [55], questions from Social Cultural Developments Dutch Sociological Questionnaire (SOCON) [58, 100]. 3. Religious coping was most commonly measured by brief- RCOPE [19, 22, 23, 25, 44, 52, 53, 101]. Other measures included Children’s Religious Coping Scale [21], Coping Orientations to Problems Experienced scale [43]. Religious Strain Scale (which measured spiritual struggle) [60], and Ways of Religious Coping (WORCS) [67]. 4. Spiritual wellbeing was measured by the Spiritual Wellbeing Scale [102], the Spiritual Involvement and Beliefs Scale [103], Spirituality Index of Wellbeing (life scheme and self-efficacy) [61, 104], Index of Core Spiritual Experience (INSPIRIT) [47], Religious Comfort Scale [43], Spiritual Meaning Scale [22], Spiritual Transcendence Index [40], Spiritual and Wellbeing Scale [85], Spiritual Involvement and Beliefs Scale [85], and King’s Spiritual Intelligence Scale [72].
Multiple aspects of religiosity and spirituality were measured by scales such as Age Universal Intrinsic-Extrinsic Scale [32, 49], Duke University Religion Index [35, 36], Fetzer Brief Multidimensional Measure of Religiousness/Spirituality (BMMRS) [22, 24, 32, 55, 56], and short form-Francis Attitudes toward Christianity Scale [33, 34, 38], The Family Environment Scale was used in a single study to measure aspects of religious beliefs in the family [20].

**Table 4:** Measures of depression and anxiety

Measures of depression included the Beck Depression Inventory (BDI) [86], Behavioural Assessment System for Children-Second Edition (BASC-2) [52], Brief Symptom Inventory (Global Severity Index) [20], Centre for Epidemiologic Studies–Depression Scale (CES-D) [26, 59–61], Child Depression Inventory, short version (CDI-S) [28], Children’s Depression Rating Scale Revised (CDRS-R) [85], Depressogenic Inferential Style Cognitive Style Questionnaire [22], Hamilton Depression Rating Scale (HAM-D) [85], American College Health Association’s (ACHA) National College Health Assessment (NCHA) [23],Patient Health Questionnaire (PHQ) [82], Schedule for Affective Disorders and Schizophrenia for School-Age Children-Epidemiologic version (K- SADS-E) [36, 43].
Measures of anxiety included Anxiety Disorders Interview Schedule (ADIS) [67], Beck Anxiety Inventory (BAI) [79], Generalized Anxiety Disorder questionnaire (GAD-7) [82], Impact of Events Scale (IES) [60], Kendall, Henin, Macdonald, and Treadwell's anxiety scale [54], Breslau’s screening scale for PTSD symptoms [27].
Measures of both depression and anxiety included the Depression, Anxiety and Stress Scale (DASS) [53], the Goldberg General Health Questionnaire (GHQ) (depression and anxiety subscales) [33], the Positive and Negative Affect Scales (PANAS) [40], Youth Self Report (ages 11–18 years) (YSR) and Adult Self-Report (ASR) of Achenbach System of Empirically Based Assessment (ASEBA) [58], National Institute of Mental Health Diagnostic Interview Schedule for Children IV (NIMH-DISC-IV) [50] and the Outcome Questionnaire (OQ-45) anxiety, depression and substance use subscale [87].

Where studies reported multiple effects for the same construct (e.g., frequency of meditation/ prayer and religious importance), these were combined into one effect size using CMA. Where studies reported an effect size for more than one independent subgroup in their sample (e.g., males and females), we included each subgroup as a separate ‘study’. Adjusted and unadjusted effects were both extracted where reported, with the primary analysis focused on adjusted effects in preference to unadjusted [105]. Meta-analyses of unadjusted effects were conducted as supplementary analyses to explore the impact of confounding. Separate meta-analyses were conducted on the four constructs of interest: religious salience, negative religious coping, positive religious coping, and spiritual wellbeing. As studies using measures of church attendance as the only indicator of spirituality or religiosity were excluded, we could not conduct a meta-analysis for engagement in organisational and formal religious practices.

The effect of gender and the presence of a stressor or risk factor were explored in separate meta-analyses where possible. When interpreting mean effect sizes, we followed Cohen's guidelines whereby r value of 0.1 = small, 0.3 = medium, and 0.5 = large [106].

Statistical heterogeneity was examined with the I^2^ statistic, which expresses the amount of heterogeneity in effect sizes in percentages. A percentage of 25% indicates low heterogeneity, 50% moderate and 75% high heterogeneity [105].

Small study effects (e.g. publication bias) were assessed when there were 10 or more studies in the meta-analysis by visually examining the funnel plot, supplemented by Egger's test of funnel plot asymmetry [107].

Intervention study findings were synthesised narratively. Where data were available, we reported differences in outcomes according to race, ethnicity, or gender and evaluated findings by these groupings (See Appendix 2). We also reported on the moderating role of these beliefs in at-risk groups (See Appendix 3, 4, 5).

### Consultations

The review was informed by consultations with young people. Focus group discussions were conducted with two cohorts of lived experience consultants: young members of local faith groups based in Delhi, India who identified as having used their religious and spiritual beliefs to overcome life-challenges, *n* = 8, all males, and young people with lived experience of anxiety and/or depression based in Mumbai, India *n* = 4, 2 males, 2 females. Participants were invited by distributing an information pamphlet to local faith groups.

In addition, one-on-one interviews were conducted with religious leaders with over 30 years of experience in leading worship and providing guidance and instruction to young members of their communities (*n* = 2). One of the leaders was based in a rural village in Uttar Pradesh and the other one was based in Mumbai. Informed consent was obtained from all participants.

The focus group discussions and interviews were semi-structured and informed by a discussion guide developed by the research group. The guide was designed to elicit opinions on young peoples’ definitions of religion and spirituality, and their use of religion and spirituality in the context of adversity and recovery/remission when experiencing depression or anxiety. A final draft of the search terms for the systematic review was finalised in discussion with the consultants during group discussions. Discussions were conducted either in Hindi (local language) or English and facilitated by the first author (SA). Reflections from the group discussions and interviews were used in various aspects of the systematic review, including to assist in the interpretation of the results.

## Findings

Overall, 74 studies (45 longitudinal and 29 intervention) met our inclusion criteria (Fig. 1). See Table 1 for longitudinal study characteristics, Table 2 for intervention study characteristics, and Appendix 2 for a summary of region, age, gender, and quality distribution.

### Longitudinal studies

Out of 45 longitudinal studies, 31 assessed engagement in religious and spiritual practices and also included measurement of at least one other aspect of religiosity or spirituality. Another 21 studies measured religious and spiritual salience, 13 studies measured religious coping, and spiritual wellbeing was measured in three studies.

#### Religious and spiritual practices

Longitudinal studies most commonly looked at the impact of religious and spiritual practices and on depression. 15 out of 31 longitudinal studies that included a measure of practices found these practices to be protective against depression; a single study found the protective effects to be restricted to females; a reverse association was found in two studies (decrease in depressive symptoms led to increased religious participation), and an increase in religious practices was associated with increased depressive symptoms in three studies (Table 5).

**Table 5:**
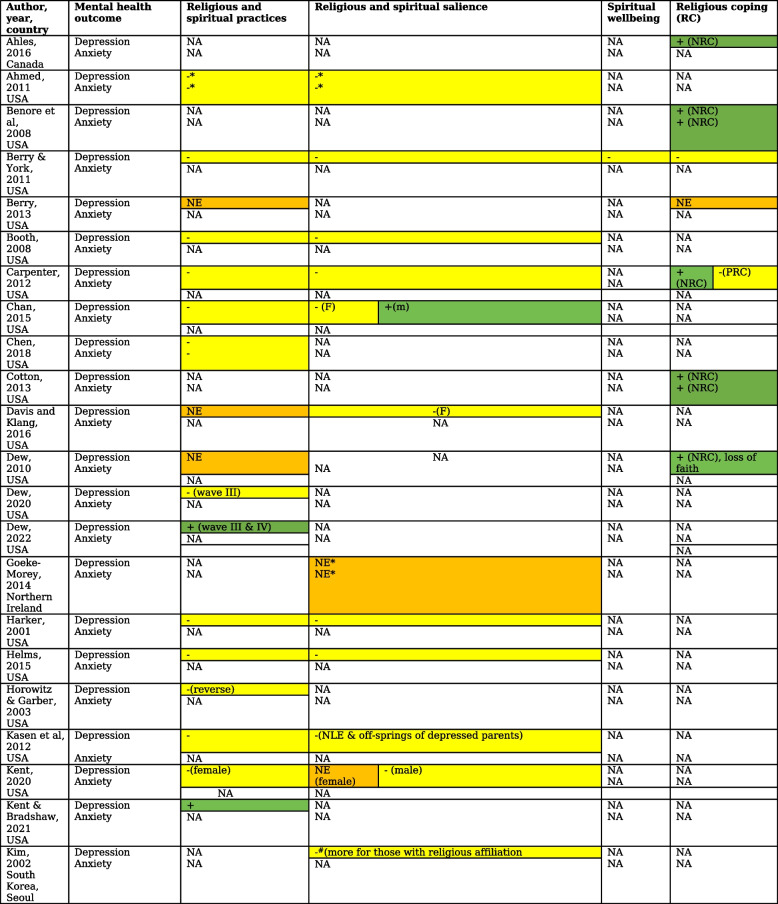

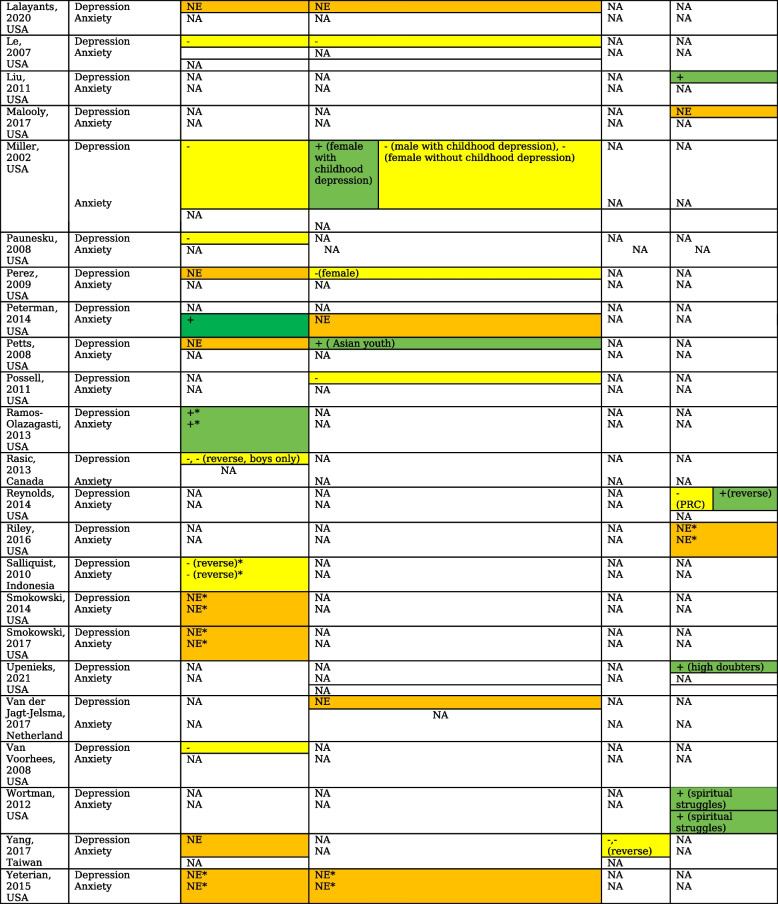
Relationship of aspects of religiosity and spirituality with depression and anxiety [6, 19–44, 46–61, 94, 108]

NA not available. F female, m- ethnic minority status, NLE negative life event, NRC negative religious coping, PRC positive religious coping, NE no statistically significant effect, * depressive and anxiety symptoms measured as emotional and behavioural problems or internalizing symptoms,# negative affect on PANAS, reverse- presence/absence of depression/ anxiety influencing religious/spirituality

Protective effect  No effect  Exacerbating effect

Out of 15 studies that found protective effects of religious practices in depression, three used Add Health data. One study reporting on the first two waves of data from Add Health found that a low level of participation in religious activities led to a significant reduction in the Population Attributable Risk (PAR) of new-onset depressive episodes in adolescents in the US over a year [24]. The effect of participation in organised religious activities such as youth groups was higher than the religious salience in this study (PAR 36% versus 13%). Another study using the same data found religious activities such as weekly prayer, describing oneself as an adherent follower of an organized religion, and attendance at a religious youth group, strongly protective against a new episode of depression [59]. In a study that used data from all four waves of Add Health over 13 years religious attendance (measure of practice) was protective for both gender [38].

Another high-quality study showed an association between greater average religious participation in practices with lower average depressive symptoms over four years of transition from adolescence to young adulthood [26]. The findings were not supported by a moderate-quality study that found no relationships between positive ecological transactions enhancing religious orientation and internalising problems over 3 years in a large cohort of middle school students [55].

Seven out of 12 studies that assessed interactions between gender, religiosity and spirituality, and the impact on the course of depression and anxiety were high in quality (See Appendix 4). Of these, two studies found participation in religious and spiritual activities to be protective against depression in both genders [26, 51]. Four high-quality US studies explored the role of race and ethnicity in the relationship between religiosity and spirituality and depression [26, 29, 48, 50]. Of these, three high-quality studies found participation in religious and spiritual practices to be associated with increased depressive symptoms in ethnic minorities, while a single high-quality study found no significant effect (See Appendix 4).

The relationship between religiosity and depression in offspring of parents with depression was assessed in two high-quality studies. One found religious practices to be protective against depression whereas the other found a reverse association [36, 37].

Of three high-quality studies that assessed the links between spirituality and religion with anxiety, one showed an association of increased anxiety symptoms with greater religious participation over a 5-year period spanning early to mid-adolescence. These findings were similar to another study (Boricua Youth Study) of Puerto Rican youth who had migrated to New York, which found participation in religious activities worsened anxiety symptoms over four years [7, 50]. A single study found weekly service attendance to be protective against post-traumatic stress disorder [27].

Some studies (k = 7) assessed anxiety symptoms as part of internalizing disorders with the majority (k = 6) not finding any protective effect of participation in religious and spiritual activities or religious coping [33, 53–56, 94]. The sole (moderate-quality) longitudinal study from a LMIC, which involved Indonesian Muslim adolescents, used a measure of religious and spiritual practices and found no effect on internalizing symptoms at one-year follow-up [54].

#### Religious salience

Nine out of 21 longitudinal studies that assessed association between religious salience and depressive symptoms found protective effects (See Table 6). Four (two high- and two moderate-quality) of these reported on data from Add Health, a representative sample study of US adolescents spanning 13 years and four waves [24, 34, 38, 42].

**Table 6:** Lived experience consultants on the role of religious and spiritual beliefs in their lives

***Religious and spiritual beliefs a way of life*** Lived experience consultants felt that spirituality was central to their way of life. Spirituality influenced their dietary and lifestyle choices making these healthier and allowed mastery of various aspects of their lives. They talked about all aspects of life of a person holding spiritual and religious beliefs being influenced by these One lived experience consultant defined beliefs as: “A way of life that determines our lifestyle choices, how we connect with ourselves and other people, and our reactions in adversities.” 22 years, male “Spirituality and religious beliefs are a way of life for us. We have an alternate name given to us by religious guru by which we are known in the religious community and that is our identity.” 23 years, male “Our life is very different from those who don’t hold these beliefs, we are kinder to others, don’t use substances such as alcohol and cannabis that many young people use". 18 years, male
***A way to connect with like-minded people*** Lived experience consultants spoke about how the beliefs help them connect with other people that share their values. They talked about people belonging to religious community acting as a biggest support system for them. “We know we are there for each other if we need anything. The world is a selfish place where no one does anything without expecting something in return. Whereas I know I can rely on my religious community brothers in crisis without the fear of being cheated and taken advantage of.” 22 years, male
***A panacea for all adversities*** Young people stressed the importance of feeling watched over and of spirituality serving as a guiding light in difficult times. A small number talked about questioning the practices that the family members wanted young people to use when they were going through a difficult time. However, they felt that most often young people were able to find something that worked for them “My mother wants me to pray when anxious. It doesn’t help and I can’t focus. I find it really hard to pray or go to place of worship in that state.” 18 years, female An ability to accept life as it is, was helpful at these times. A reminder of it being a passing phase, the feeling of someone watching over them and ability to see the whole picture (e.g., how trivial these problems were in the larger scheme of things) were crucial at these times to allow them to accept life challenges “Anything that happens in my life, is for a reason. I know that someone knows the plan and will see to it that I come out of it unscathed.” 22 years, male
***Spirituality versus religiosity*** Young consultants felt the distinction between spirituality and religion was arbitrary. They reflected that even though practices such as chanting and praying are counted as religious, these allowed them a reflective space to understand themselves better and thus could be considered as spiritual when used in this way “I connect with myself when I pray. I am able to think clearly and understand my thoughts better.” 18 years, female “I like the feeling of being watched over especially in times of difficulties. It helps me in not to lose hope.” 24 years, male
***Evolution of beliefs over time*** “My dad is an atheist and I really admire his strength. When my mental health problems started, I used to pray a lot. It didn’t help. Nothing got better. I got help from people around me who suggested how to deal with the problems. I practiced meditation, breathing exercises which was helpful. My beliefs have evolved now, I feel I can rely on people around me for help rather than a lifeless idol.” 19 years, female

**Fig. 2:**
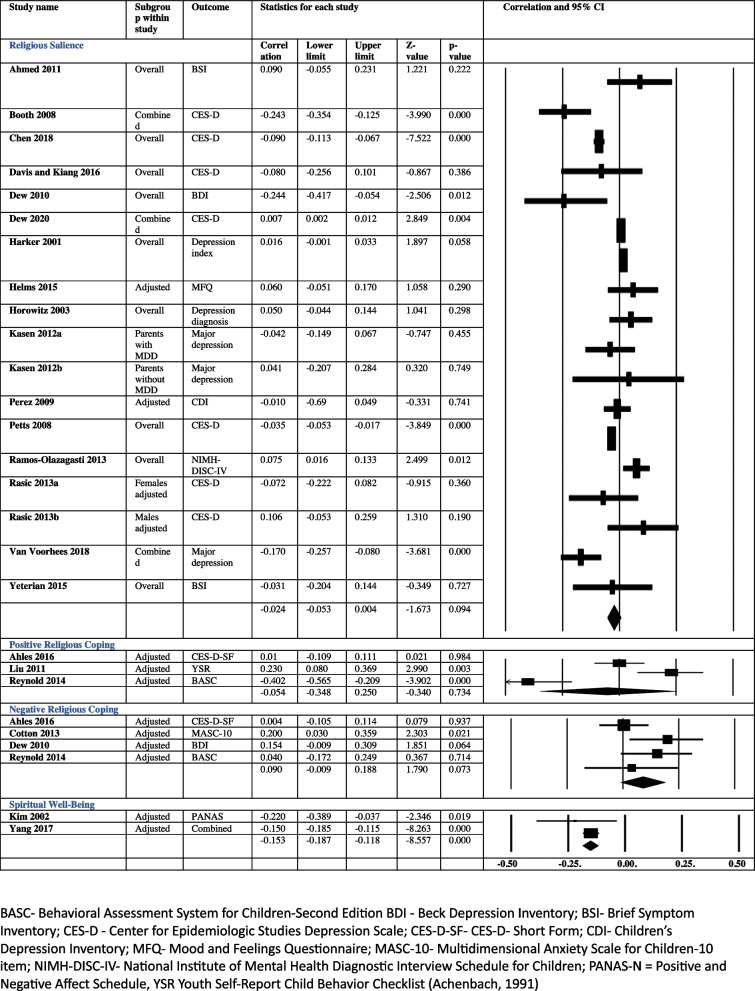
Forest plot visualizing the impact of religious salience, negative religious coping, and spiritual wellbeing in depressive symptoms. BASC- Behavioral Assessment System for Children-Second Edition BDI - Beck Depression Inventory; BSI- Brief Symptom Inventory; CES-D - Center for Epidemiologic Studies Depression Scale; CES-D-SF- CES-D- Short Form; CDI- Children’s Depression Inventory; MFQ- Mood and Feelings Questionnaire; MASC-10- Multidimensional Anxiety Scale for Children-10 item; NIMH-DISC-IV- National Institute of Mental Health Diagnostic Interview Schedule for Children; PANAS-N = Positive and Negative Affect Schedule, YSR Youth Self-Report Child Behavior Checklist [63]

We pooled the effects across 16 high-quality longitudinal studies (18 samples) from which data for religious salience was available. Pooling did not show a significant effect of religious salience on depressive symptoms (*r* = -0.024 [-0.053, 0.004], *p* = 0.094) [29] (Fig. 2). There was a large heterogeneity between the studies (I^2^ = 87.2%) and no evidence of publication bias (Egger's test, two-tailed *p* = 0.184). Longer follow-ups were associated with diminishing effect of religious salience on depressive symptoms (Fisher’s z score = 0.0015, CI 0.0005, 0.0026, *p* = 0.005).

**Fig. 3:**
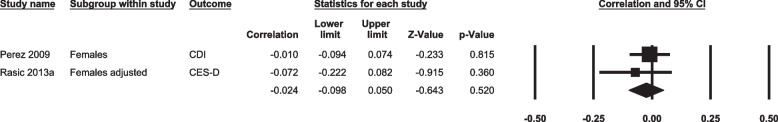
Forest plot visualizing the impact in females of religious salience on depression

In a moderate-quality study, religious salience in women with history of childhood depression was linked to depression in adulthood whereas in men, religious affiliation protected against depression in adulthood [45]. Three studies found protective effects of religious salience in females but not in males [26, 29, 47]. The pooled effect in females of religious salience on depression across two high-quality studies was not significant *r* = -0.024 [-0.098, 0.050], *p* = 0.520 [47, 51] (Fig. 3). The only high-quality study that looked at gender differences in the association of religiosity with anxiety showed no differences in outcomes [6].

The links between religious salience and anxiety and depression were compared among faith groups and religious denominations in five studies [6, 23, 36, 58, 108]. A single moderate quality study showed protective effects of Catholic denomination of Christian faith as compared to Protestant denomination in males with history of childhood depression [45]. Ethnicity or religious denomination did not interact with religious salience and practices over five years from early- to mid-adolescence to predict anxiety in a single high quality study [6].

Among high-risk groups, religious salience and practices offered protection against emotional and behavioural problems in homeless youth and those with physical and relational victimization in two high-quality studies whereas there was no association in youth involved with child welfare services in a moderate-quality study [20, 35, 41]. In yet another moderate-quality study, religious salience and church attendance did not influence the relationship of psychiatric problems during pre-adolescence and internalising problems during adolescence [58].

#### Religious coping

Six (three high-quality and three moderate-quality) out of ten studies that assessed the moderating role of religious coping with various risk factors found negative religious coping to increase the risk of depression and anxiety [19, 21, 25, 28, 43, 60]. A single high quality study that assessed direct relationship between religious coping and depression found loss of faith predicted less improvement in depressive symptoms over six months [32]. Pooling of adjusted effects across four high-quality studies (three studies that assessed moderating role and a single study assessing direct effect of religious coping) showed a trend for increased risk of depressive symptoms with negative religious coping, with an overall effect size of *r* = 0.09 [-0.009, 0.188], *p* = 0.073 and low levels of heterogeneity (I^2^ = 36%) [19, 28, 32, 52]. Supplementary analysis with unadjusted values across two studies showed a bigger effect size *r* = 0.17 [0.079, 0.259], *p* < 0.001 [19, 101]. Pooling of adjusted effects across three high-quality studies did not show a significant effect of positive religious coping on depressive symptoms (effect size *r* = -0.054 [-0.348, 0.250], *p* = 0.734 and high levels of heterogeneity, I^2^ = 91.7%) [19, 43, 52] (See Fig. 2 and Appendix 1). The role of religious coping in mediating the relationships between gender and depressive symptomatology was assessed in a single high-quality study with no significant findings [44].

#### Spiritual wellbeing

Three studies examined the impact of spiritual wellbeing as a process on depression [22, 40, 61]. Pooling the effect of spiritual wellbeing on depressive symptoms across two high-quality studies showed protective effects with an overall effect size of *r* = -0.153 [-0.187, -0.118], *p* < 0.001 and no heterogeneity [40, 61] (see Fig. 2). A third moderate-quality study found baseline spiritual meaning was negatively and moderately correlated with stress and depression at 6-month follow-up in a cohort of University students [22].

### Intervention studies

The types of interventions evaluated in included studies varied. Multi-session interventions that incorporated education with opportunities to practice spiritual techniques such as meditation, yoga and prayer were the most common type investigated for their impact on depression and anxiety symptoms in young people. Other interventions aimed to integrate spiritual or religious components into therapeutic interventions. Interventions were delivered online (k = 2), to individuals (k = 5) or to groups (k = 22). Most universal interventions were group-based, and with limited exceptions, most of these reported beneficial effects on anxiety and depression symptoms. However, only one of the studies that evaluated a universal group intervention was an RCT, limiting confidence in the findings.

There was a significant overlap in the dimensions of religious and spiritual beliefs that were used in the intervention studies. However, 12 studies used predominantly religious and spiritual practices in interventions (two high-quality studies), seven studies (no high-quality studies) used interventions that tapped into religious salience, and ten studies (six high-quality studies) used predominantly spiritual wellbeing enhancement as a process to improve depression and anxiety symptoms in young people.

#### Religious and spiritual practices

In a high-quality study with University students with mild to moderate depressive and anxiety symptoms, significant reductions in both were observed after seven weeks of bi-weekly sessions of Hatha Yoga and meditation [89]. Similar decrease in anxiety levels post-intervention were observed in another high-quality study, during which the participants were taught practices based on Buddhist meditation traditions over 13 weeks [84].

A moderate-quality RCT of behavioural activation of religious behaviours (BARB) in 50 Canadian US college students scoring above a cut-off on the BDI showed that, compared to supportive therapy, one 60-min session of behavioural activation led to statistically significant decreases in depression, somatic and trait anxiety with maintenance of effects at 1-month follow up [67]. Religious behaviours and spiritual wellbeing significantly mediated the relation between treatment condition and depression severity.

#### Religious salience

In a moderate quality study, Hajra and colleagues used daily Islamic art therapy sessions for 14 days in a group of University students with mild to moderate anxiety and depressive symptoms [74]. There was a significant decrease in both depression and anxiety scores at post-intervention assessment. Two other moderate quality studies that harnessed personal importance of religion during counselling sessions found improvements in anxiety and depressive symptoms [76, 77]. Chen and colleagues in a series of three RCTs (low-quality) used religious perspective for trauma processing as an intervention. In two studies there was no significant effect on post-traumatic stress disorder symptoms whereas in the third study there was a significant reduction in depressive symptoms post-intervention as compared to the control group [69–71].

#### Spiritual wellbeing

Evidence was stronger for spiritual wellbeing related interventions targeted to adolescents with higher levels of depression and anxiety symptoms.

In a high-quality RCT with a waitlist control design, Rickhi et al. evaluated the effectiveness of an online spirituality informed e-mental health intervention that guided participants through an exploration of spiritually informed principles (e.g. forgiveness, gratitude and compassion) in 62 Canadian adolescents (aged 13–18 years) and young adults (19–24 years) with mild to moderate major depressive disorder [85]. Impact of 8 weekly online modules on depression severity, spiritual wellbeing and self-concept was measured at eight, 16 and 24-week follow-up with a significant reduction in depressive symptoms at post-intervention in the online intervention group compared to the control group and maintenance of effect in the younger subgroup at 16 and 24 weeks. In another high-quality RCT, Pandya and colleagues assessed the impact of an online spiritual counselling program (50 one-hour weekly sessions) in mitigating anxiety, and building self-esteem and academic self-efficacy in 96 deaf and hard-of-hearing students in India, Kenya, Nepal and South Africa [83]. Compared to participants in a control intervention (online relaxation sessions), intervention participants had significantly lower anxiety scores post-intervention.

In two RCTs (moderate and high risk of bias respectively), Wachholtz and Pargament examined whether meditation with an explicit focus a participant’s spiritual belief system was more effective than secular meditation and relaxation in improving mental health outcomes [91, 109]. In one study, participants were 92 college students who had not previously practiced meditation and who met the criteria for vascular headache [91]. Compared to secular meditation and relaxation groups, the spiritual meditation group had greater decreases in trait anxiety and negative affect. There were no significant differences between groups in depression scores. In the other study [109], which involved 84 students without identified health conditions, students in the spiritual meditation group reported a greater decline in anxiety symptoms than students in the other groups.

Singh and colleagues in a series of studies (k = 3) taught about the benefits of spiritual practices such as mindfulness and mediation with videos of Indian spiritual leaders to a group of University students. They found a significant decrease in depressive and anxiety symptoms after 14 intervention sessions in one of the three studies [88].

In a low-quality study conducted in Iran [75], 60 young people who had been admitted to hospital following a suicide attempt were randomly divided into two groups, one of which received a spiritual care intervention (consisting of educational booklets, face-to-face guidance and the offer of telephone support from a counsellor) while the other group received usual care. There was a significant difference in depression symptoms between the two groups post-treatment. Another low-quality trial conducted in Iran which evaluated the impact of “spiritual intelligence training” in 60 secondary students [68]. No differences in mental health outcomes were seen.

Finally, a group spirituality intervention in 122 young women in an eating disorder inpatient unit was compared with both cognitive and emotional support interventions in a three-arm RCT [87]. Women in the spirituality group had significantly greater reductions in anxiety and depression symptoms than those in the other groups.

## Discussion

A systematic review and meta-analysis of longitudinal studies investigating the relationship between aspects of religiosity and spirituality in the prevention and management of depression and anxiety in young people found that negative religious coping showed a trend towards association with greater depressive symptoms over time, whereas spiritual wellbeing was protective against depression. The effect sizes were minimal. Participation in religious practices may protect young people against depression and, to a lesser extent, anxiety, especially over shorter periods of time. Findings from intervention studies support the integration of aspects of religious and spiritual practices into preventive and treatment interventions, particularly for young people at risk or with higher symptom levels of anxiety and depression. Interventions promoting spiritual wellbeing were largely effective in reducing depressive and anxiety symptoms.

However, an overwhelming majority of studies included in the review were from high-income countries (mostly the U.S., k = 50) and used a Judeo-Christian concept of religion. Only a single longitudinal and a few (k = 15) intervention studies were from LMICs, in which 90 percent of the world’s adolescents live [54]. Although almost all studies from LMICs had moderate to high-risk of bias, we included them in the current review to highlight the scope of research in contexts that vary widely in the importance placed on religion and spirituality [110].

The importance of religion in the lives of young people residing in LMICs, and how these beliefs impact on mental health might differ from those in countries where religiosity and spirituality is a less integral part of the cultural milieu [96, 110]. For example, in the only longitudinal study from an LMIC (Indonesia, one of the top ten most religious countries) [54, 111], an experience of greater internalizing symptoms in transition from early- to mid-adolescence over a year led to an increased participation in religious activities, possibly as a socially acceptable way to get support or because of advice from community members or religious leaders. However, two high-quality US studies showed associations between increased religious participation and higher internalizing symptoms during the same age range over 4–5 years [7, 50]. It is possible that the social aspects of service attendance or any other religious gathering are challenging in early adolescence, if this is not a culturally acceptable way to get support when vulnerable (e.g. Puerto Rican migrant adolescents in New York in one study) [50, 112]. A better understanding of universal versus culturally specific elements of religious and spiritual trajectories and processes can occur by evaluating both the expression of the beliefs as well as the purpose they serve in diverse contexts [113].

Findings differed according to the length of time for which the relationship between the beliefs and outcomes was observed. Many studies that showed helpful effects had a follow-up duration of anywhere between four months to a year and the participants were mostly in mid-adolescence (all studies were moderate quality) [33, 42, 49]. The association changed when observed for longer periods. For example, in a high-quality study using representative data from the first two waves of Add Health, religious activities and salience were protective against depression over a year, whereas when all four waves of data over thirteen years were used, religious participation was associated with increased odds of depression (moderate-quality study) [24, 38]. Most of the 16 studies used to calculate the effect of length of follow-up in religious salience and depressive symptoms had a follow-up period of 1 to 2 years, but varied up to 20 years. This could explain the small effect sizes observed in meta-regression analyses.

The findings highlight the dynamic nature of religious beliefs and their evolution during transition to adulthood [24]. Religious participation during early adolescence could be related to familial religiosity whereas with greater cognitive development, the protective effect of religious salience may become relevant during mid- to late- adolescence [33, 49, 93]. It is thus possible that the benefits of religiosity and spirituality in the prevention and management of depression and anxiety are more pronounced at specific timepoints during adolescence. For example, the protective role of religion and spirituality appears to start in early adolescence across cultures, particularly in females, with aspects such as spiritual connectedness, sources of inspiration, and guidance explaining the effect [47, 54, 61]. Religious involvement during mid-adolescence may provide links to a community structure and access to resources (e.g. positive role models) that can shape a young person’s evolving sense of identity, and help them make mental health promoting lifestyle choices such as avoiding substances [114]. Our findings are similar in direction, but weaker in strength, to the findings observed in a recent systematic review of prospective studies across lifespan (152 studies). The review showed a negative association between measures of religiosity and spirituality and depression (Cohen’s d = -0.18), and a positive association between religious struggles (included in negative religious coping in the current review) and depression (Cohen’s d = 0.3) [15, 16]. However, non-significant results were more often found in studies with samples with a mean age below 25 in this review [16].

The findings of the current review suggest a complex relationship between spirituality and religion, and depression. Although religious and spiritual beliefs seem to offer protection against depression during early to mid- adolescence, a depressive episode during this period could lead to feelings of insecurity, undermine a personal connection with a higher power and hinder development of religiosity and spirituality by influencing complex, personal representations of God that are formed during this crucial phase [33, 47, 54, 115]. These feelings, when combined with anger towards God, negative encounters with other members of their faith community or internal religious guilt or doubt, can make young people more vulnerable to future episodes of depression and anxiety [10]. The most consistent finding across longitudinal studies was the protective role of spiritual wellbeing in depression and anxiety. This was also reflected in the findings from intervention studies. It is possible that some of the unhelpful consequences associated with negative representation of God and internal religious guilt are bypassed when a more spiritual wellbeing-based approach using concepts such as forgiveness, gratitude, compassion, and acceptance is used.

Spiritual and religious beliefs appear to help young people cope better with stressful life circumstances and get much-needed support from the community, especially in places where religion is an integral part of life. Although the role of religion or spirituality varies according to country and culture, in many places religious leaders are often among those first sought out for advice by families of young people experiencing mental health problems [116]. This can lead to potential delays in accessing evidence-based care due to either excessive reliance on religious processes (e.g. praying) or advice from religious leaders [117]. Using well established religious platforms to promote the mental health and in the management of mental disorders is likely to help harness these beliefs to the advantage of young people. Religious leaders can be trained to disseminate evidence-based information and address stigma associated with mental health. For example, UNICEF and its partner Bangladesh Rural Advancement Committee trained more than 300 religious leaders (Imams) in the camps in Bangladesh to address stigma and dispel misinformation about COVID-19, pointing to the value of partnership with religious leaders in healthcare [118]. Furthermore, religious and spiritual beliefs should be explored in mental health assessments in youth and used in treatment plans where appropriate. The World Psychiatric Association recommends an understanding of religion and spirituality and their relationship to the diagnosis, aetiology and treatment of psychiatric disorders as essential components of both psychiatric training and continuing professional development [119].

### Insights into mechanisms

**Fig. 4:**
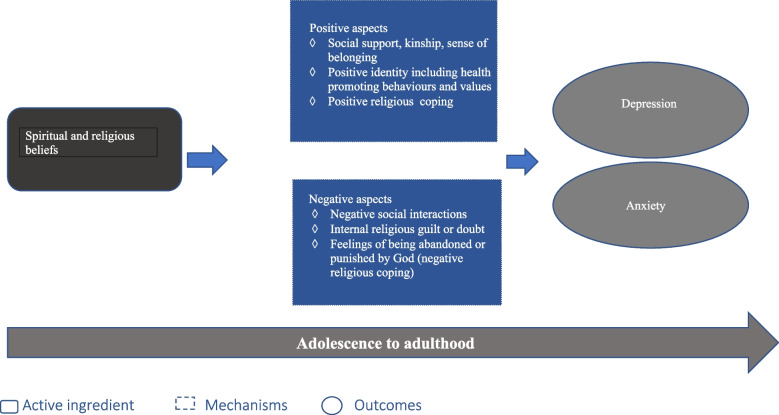
Mechanism of action

Drawing upon the findings of the studies in the review and inputs from the lived experience consultants, we propose a logic model for the mechanisms through which religious and spiritual beliefs might have an impact in depression and anxiety in young people (Fig. 4).

Religion and spirituality can have a beneficial impact by allowing the development of a s positive identity, providing opportunities to connect socially, and offering a way to cope with difficulties. Aspects of positive identity such as self-efficacy and self-esteem mediated the effect of religious and spiritual beliefs in depression and anxiety in five studies while in the sixth study, mediating effects were restricted to females [29, 47, 48, 51, 54, 61]. Lived experience consultants spoke about the influence of these beliefs on their sense of identity, values, behaviours and competencies in ways they considered mental health promoting. Positive religious coping offered protection in two moderate quality studies. This aspect was highlighted by one of the religious leaders who suggested that the beliefs lent an ability to look at the bigger picture and not getting invested emotionally in inconsequential things in life (See Table 7). Social support was one of the key mediators in two studies [34, 48], and to a lesser extent in another two studies [32, 51]. This was explained by the consultants as a feeling of kinship, a sense of belonging to a group of like-minded people who they could trust, and who shared their beliefs and values (See Table 6).

**Table 7:** Religious leaders on role of spiritual and religious beliefs in depression and anxiety in young people

***Role of spirituality during adolescence- malleable age*** According to religious leaders, 14 to 24 years age range is very crucial in understanding spirituality. Spirituality if used at this age could drastically transform the lives of young people. They felt that the life decisions taken at this time have long term repercussions. Spirituality can act as a guiding path to allow young people to make right choices about their lifestyle and future. It has the potential to make them focused and lend meaning to their lives. For youth, who are disadvantaged and experienced disruption in attachment with caregivers, spirituality could offer a reparative experience and allow them to achieve their potential.
***Spirituality as a way to live mindfully*** Another important aspect of spirituality highlighted by the leaders was the ability to live mindfully in the present moment without fears about future and regret about the past. This aspect was related to acceptance that young people spoke. Religious leaders talked about an attitude of nonchalance arising from the feeling of being watched over resulting in mindful living.
***Spirituality and emotional regulation*** According to the leaders, spirituality could improve control over emotions such as anger and control mood swings. They spoke about the beliefs lending an ability to look at the bigger picture and not get invested emotionally in inconsequential things in life.
***Altruism as an extension of spirituality*** One of the leaders spoke about how altruistic behaviour is promoted by spirituality as a result of which young people can experience happiness. He suggested that the core of spirituality and religious beliefs is that “I am one with others” and “my happiness is not independent of what others are going through”. Someone who truly believes in this would help others and try doing good for the society as it is an extension of one’s own self.
***Role of familial spiritual and religious beliefs*** Leaders highlighted the role of familial beliefs in the belief system of adolescents. They talked about how young people’s own beliefs are related to what they observe being practiced from early on in their lives. One of the leaders spoke about the emotional regulation skills of the mother/ primary caregiver and their responses in the time of crisis getting imbibed by the children by observational learning. “If they see their parents behaving calmly and using a particular set of practices to overcome the challenges, they are likely to use the same”.
***Spiritual education*** Spiritual leaders spoke about the importance of introducing spirituality in the education system as it helps in personal development and gives tools to young people to deal with life challenges. One of the leaders talked about how he had been running a school in the rural area of India where the children are from disadvantaged different religious backgrounds (Hindu, Muslim, Christian). They promote spirituality in this context by going beyond various religious denominations, teach them meditation and how to be more considerate towards others.

On the other hand, negative religious interactions, internal religious guilt and negative religious coping are likely to exacerbate depressive and anxiety symptoms. Negative religious coping exacerbated the effects of stress and physical illness leading to internalising disorders in five studies (three high quality) [19, 21, 22, 25, 43, 52, 101]. Figure 4 presents the mechanisms through which spiritual and religious beliefs can have an impact on depression and anxiety.

### Strengths and limitations

Strengths of the review include a focus on the aspects of religious and spiritual beliefs beyond just religious attendance as well as the inclusion of studies from a broad range of settings, including LMICs in which religion and spiritual beliefs are key cultural influences. To the best of our knowledge, this is the first comprehensive systematic review of longitudinal and intervention studies that assesses the role of different aspects of spiritual and religious beliefs in prevention and management of depression and anxiety in young people.

Key limitations of the evidence base include the very limited number of longitudinal studies from LMICs, low quality of many studies (particularly the intervention studies), less rigorous research designs, non-representative small samples (aside from three representative studies from the US) and short follow-up durations. Most of the intervention studies were conducted in college students with no pre- and post-assessment of religious or spiritual beliefs.

Future research should focus on the incorporation of standardized definitions of key constructs across studies and move beyond single-item measures of religious service attendance by considering both distal domains of religion/spirituality (behaviours such as frequency of service attendance), and proximal domains (functions of religion/ spirituality such as spiritual support, religious coping (including negative religious coping), spiritual meaning and influence of religion and spirituality on identity, competencies, values and behaviours. This may assist in reaching a consensus about domains that are related to internalizing disorders and may also guide the development of interventions [101, 120].

## Conclusion

Spiritual wellbeing can offer protection against depressive symptoms whereas negative religious coping can exacerbate effects of stress and lead to increased risk of depression. Participation in religious and spiritual activities in adolescence and young adulthood can have short- to medium- term protective effects in depression and to a lesser extent in anxiety. Interventions based on spirituality, and psychotherapies that integrate these beliefs in the management of depression and anxiety in young people, may be beneficial.

Although limitations in the evidence base limit our ability to draw definitive conclusions, the review helps us understand the ways the beliefs can offer support to young people. Introducing concepts of spirituality including self-efficacy and life meaning during early adolescence can help the development of a young person’s ‘meaning making system’, influence their sense of identity and lifestyle choices, and can prepare young people for the life challenges during mid- to late- adolescence.

Additionally, participation in religious activities when young people consider religion to be salient, can offer support during this developmental phase. These measures should be combined with guidance about having clear goals in life and promoting self-reliance [121]. There is a pressing need for research in regions where religion and spirituality are an integral part of life for young people. This may include further exploration of the role of religious leaders in promoting mental health and exploring novel ways to harness these beliefs and minimising the negative impacts of religion to assist in preventing and managing depression and anxiety.

### Supplementary Information

**Additional file 1:** **Appendix 1.** Search terms for identifying eligible studies. **Appendix 2.** Region, age, gender, quality distribution of longitudinal and intervention studies. **Appendix 3.** Risk of bias. **Appendix 4.** Gender differences in associations between spirituality and religiosity with depression and anxiety. **Appendix 5.** Race, ethnicity and faith differences in association of spirituality and religiosity with depression and anxiety. **Appendix 6.** Spirituality/ Religiosity as a moderator between risk factor and depression/anxiety.

## Data Availability

All data generated or analysed during this study are included in this published article [and its supplementary information files].
