# Supplementary material for: Religiosity and spirituality in the prevention and management of depression and anxiety in young people: a systematic review and meta-analysis
Source: BMC Psychiatry. 2023 Oct 10;23:729. doi: 10.1186/s12888-023-05091-2 (PMC10563335; doi:10.1186/s12888-023-05091-2)
Supplement: Supplementary file 1 — Additional file 1: Appendix 1. Search terms for identifying eligible studies. Appendix 2. Region, age, gender, quality distribution of longitudinal and intervention studies. Appendix 3. Risk of bias. Appendix 4. Gender differences in associations between spirituality and religiosity with depression and anxiety. Appendix 5. Race, ethnicity and faith differences in association of spirituality and religiosity with depression and anxiety. Appendix 6. Spirituality/ Religiosity as a moderator between risk factor and depression/anxiety. [file 12888_2023_5091_MOESM1_ESM.docx]

# Supplementary material

## Appendix 1: Search terms for identifying eligible studies

Search terms for prospective observational and intervention studies in Medline, PsycINFO, and Scopus are included below. All searches were conducted on July 22^nd^, 2021.

### Ovid MEDLINE

1. exp religion/ or exp spirituality/ or (religio* or spiritual*).tw,kf. or (buddhis* or islam* or hindu* or taois* or judaism).tw,kf.

2. adolescent/ or students/ or young adult/ or (adolesc* or youth* or teen* or student*).tw,kf. or ((young or emerging) adj (adult* or people or person*)).tw,kf.

3. exp depression/ or exp depressive disorder/ or (depress* or dysthymi*).tw,kf.

4. exp anxiety/ or exp anxiety disorders/ or (anxiety or agoraphobia or "panic disorder*" or "phobic disorder*" or phobia*).tw,kf.

5. psychological distress/ or stress, psychological/ or ("psychological distress" or "emotional distress" or "mental distress").tw,kf.

6. ("common mental disorder*" or "common mental health problem*" or (internali* adj (symptom* or disorder* or problem*))).tw,kf. 8505

7. "trauma and stressor related disorders"/ or adjustment disorders/ or psychological trauma/ or stress disorders, post-traumatic/ or stress disorders, traumatic, acute/ or (adjustment-disorder* or psychological-trauma or post-traumatic-stress or PTSD or acute-stress-disorder*).tw,kf.

8. obsessive behavior/ or obsessive-compulsive disorder/ or (obsessive-behav* or obsessive-compulsive-disorder*).tw,kf.

9. 3 or 4 or 5 or 6 or 7 or 8

10. 1 and 2 and 9

11. limit 10 to (english language and yr="2000 -Current")

12. limit 11 to (comment or editorial or letter)

13. 11 not 12

### Ovid PsycINFO

1. exp spirituality/ or exp religion/ or (religio* or spiritual*).tw. or (buddhis* or islam* or hindu* or taois* or judaism).tw.

2. exp adolescent development/ or exp adolescent psychology/ or exp students/ or (adolesc* or youth* or teen* or student*).tw. or ((young or emerging) adj (adult* or people or person*)).tw.

3. exp Anxiety Disorders/ or exp Anxiety/ or (anxiety or agoraphobia or panic-disorder* or phobic-disorder* or phobia*).tw. 4. exp major depression/ or (depress* or dysthymi*).tw. or "depression (emotion)"/

5. ((internali* adj (symptom* or disorder* or problem*)) or ("common mental health problem*" or "common mental disorder*")).tw.

6. exp psychological stress/ or exp distress/ or (psychological distress or emotional distress or mental distress).tw.

7. "stress and trauma related disorders"/ or acute stress disorder/ or adjustment disorders/ or exp posttraumatic stress disorder/ or post-traumatic stress/ or (adjustment-disorder* or psychological-trauma or post-traumatic-stress or PTSD or acute-stress-disorder*).tw.

8. obsessive compulsive disorder/ or obsessions/ or ("obsessive compulsive disorder*" or "obessive-behav*").tw.

9. 3 or 4 or 5 or 6 or 7 or 8

10. 1 and 2 and 9

11. limit 10 to (peer reviewed journal and english language and yr="2000 -Current")

### Scopus

1. ( TITLE-ABS-KEY ( spiritual* OR religio* OR buddhis* OR islam* OR hindu* OR taois* OR judaism )

2. TITLE-ABS-KEY ( "anxiety" OR "depression" OR "depressive symptom*" OR "depressive disorder*" OR "dysthymi*" OR "common mental disorder*" OR "common mental health problem*" OR "psychological distress" OR "agoraphobia" OR "panic disorder*" OR "phobic disorder*" OR "phobia*" OR "emotional distress" OR "mental distress" OR "internali* symptom*" OR "internali* disorder*" OR "internali* problem*" OR "adjustment disorder*" OR "psychological trauma" OR "post traumatic stress" OR ptsd OR "acute stress disorder*" OR "obsessive behav*" OR "obsessive compulsive disorder*" )

3. (TITLE-ABS-KEY ( adolesc* OR youth* OR "young person*" OR "young people" OR "young adult*" OR "emerging adult*" OR student* OR teen* ))

4. 1 AND 2 AND 3

5. LIMITS to LANGUAGE ( english ) not INDEX ( medline ) SRCTYPE ( j ) DOCTYPE(ar) PUBYEAR > 1999

## Appendix 2. Region, age, gender, quality distribution of longitudinal and intervention studies

| Among the 45 longitudinal studies, 38 (84.4%) were from the US, with two from Canada (4.4%) (33, 118), with a single study from Indonesia (34), Netherlands (46), Northern Ireland (70), South Korea (64), and Taiwan (31) each. Only a single longitudinal study was conducted in a low- and middle- income country (LMIC) (34). All longitudinal studies had a panel design with either single or multiple contacts with the participants over the follow-up period. Three longitudinal studies recruited a nationally representative sample of adolescents in the US: National Longitudinal Study of Adolescent Health (Add Health) (24); National Study of Youth and Religion (NSYR) (28); and the National Survey of Child and Adolescent Wellbeing, which had a representative sample of child welfare-involved youth (82). Study recruitment occurred in school, tertiary education, health or social services and community settings. There was a considerable variation in the total follow- up duration, ranging from four weeks to 20 years with majority having a follow-up duration of one year or less. Baseline mean age in longitudinal studies varied from 10 to 18 years (46, 55). The mean age of the participants was less than 15 years at baseline in 13 studies. (Table 1) In 28 longitudinal studies, female participants outnumbered males, whereas ten studies had a greater number of male participants (Table 1). Gender distribution was equal in four studies (25, 32, 63, 70). Three studies did not report the gender distribution (74, 79, 80). Most studies (where reported) had racially and ethnically diverse participants (principally White, Black, Asian American, African American and European American) from multiple faiths, although Christianity was most common. In one study in Indonesian adolescents, all participants were Muslim, and in a study in Taiwanese adolescents all participants were of the Buddhist or Daoist faiths (31, 34).  Among the 29 intervention studies, 12 (38%) were from the US, 7 (24%) from India, and 6 (21%) from Iran. (Table 2) Fifteen intervention studies were conducted in LMICs. The most common type of study design was the randomised controlled trial (RCT) (12 studies). There were 8 quasi-experimental studies and 9 pre-post studies. For RCTs or quasi-experimental studies, comparison conditions were typically usual care, wait-list controls or similar interventions but without spiritual components (e.g., supportive counselling or writing about trauma). Most studies (k=21; 74%) were conducted in tertiary education institutions. Mean ages were higher in intervention studies and where reported, ranged from 14.82 to 25 years. The most common age ranges were late teens and early twenties. As in longitudinal studies, most intervention studies had a greater proportion of females than males, ranging from 14.2% to 90.4%, while two studies targeted only males and a single study had only female participants (76, 87, 113). Study quality Among longitudinal studies, risk of bias was low for 25 studies (high-quality) and moderate for 20 studies (moderate-quality). In intervention studies, risk of bias was low for three RCTs (high-quality) and five quasi-experimental or pre-post therapy studies, moderate for two RCTs (moderate-quality) and 11 pre-post therapy or quasi-experimental studies, and high for eight RCTs. Out of a total of 15 studies from LMICs, only three intervention studies had a low risk of bias (risk of bias was high for four studies and moderate for eight studies). |
| --- |

## Appendix 3. Risk of bias

**Table 1. Prospective observational studies’ risk of bias criteria and assessment**

| **Study** | **Risk of bias criteria** | | | | | | | | | | | **Risk of bias score (%)** | **Risk of bias rating** |
| --- | --- | --- | --- | --- | --- | --- | --- | --- | --- | --- | --- | --- | --- |
|  | **1** | **2** | **3** | **4** | **5** | **6** | **7** | **8** | **9** | **10** | **11** |  |  |
| Ahles, 2009 | NA | NA | ✔ | ✔ | ✔ | ✖ | ✔ | ✖ | **?** | ✔ | ✔ | 66.7 | Low |
| Ahmed, 2016 | NA | NA | ✔ | ✔ | ✔ | ✖ | ✔ | **?** | ✖ | ✔ | ✔ | 66.7 | Low |
| Benore, 2008 | NA | NA | ✔ | ✔ | ✔ | ✖ | ✔ | ✖ | ✖ | ✖ | ✔ | 55.6 | Moderate |
| Berry, 2013 | ✔ | ✔ | ✔ | ✔ | ✔ | ✖ | ✔ | ✖ | **?** | **?** | **?** | 54.5 | Moderate |
| Berry & York, 2011 | NA | NA | ✔ | ✔ | ✔ | ✖ | ✔ | ✖ | ✖ | ✖ | ✔ | 55.6 | Moderate |
| Booth, 2008 | NA | NA | **?** | ✔ | ✔ | ✔ | ✔ | ✔ | ✖ | ✔ | ✔ | 77.8 | Low |
| Carpenter, 2012 | NA | NA | ✔ | ✖ | ✖ | ✖ | ✔ | ✖ | **?** | ✔ | ✔ | 44.4 | Moderate |
| Chan, 2014 | NA | NA | **?** | ✔ | ✔ | ✖ | ✔ | ✔ | ✔ | ✔ | ✔ | 77.8 | Low |
| Chen, 2018 | NA | NA | ✖ | ✔ | ✔ | ✖ | ✔ | ✔ | ✔ | ✖ | ✔ | 66.7 | Low |
| Cotton, 2013 | NA | NA | ✔ | ✔ | ✔ | ✖ | ✔ | ✔ | ✔ | ✔ | ✔ | 88.9 | Low |
| Davis & Kiang, 2016 | NA | NA | **?** | ✔ | ✔ | ✖ | ✔ | ✔ | ✔ | ✔ | ✔ | 77.7 | Low |
| Dew, Fuemmeler, & Koenig, 2020 | NA | NA | **?** | ✔ | ✔ | ✖ | ✔ | ✔ | NA | NA | ✔ | 71.4 | Low |
| Dew, Kollins & Koenig, 2020 | NA | NA | **?** | ✔ | ✔ | ✖ | ✔ | ✔ | NA | NA | ✔ | 57.1 | Moderate |
| Dew, 2010 | NA | NA | ✔ | ✔ | ✔ | ✖ | ✔ | ✖ | ✔ | ✖ | ✔ | 66.7 | Low |
| Goeke-Morey et al, 2014 | NA | NA | ✔ | ✔ | ✔ | ✖ | ✔ | ✔ | NA | NA | ✔ | 57.1 | Moderate |
| Harker, 2001 | ✔ | ✔ | ✖ | ✔ | ✔ | **?** | ✔ | ✔ | NA | NA | ✔ | 77.8 | Low |
| Helm, 2015 | NA | NA | ✔ | ✖ | ✖ | **?** | ✔ | ✔ | ✔ | ✔ | ✔ | 66.7 | Low |
| Horowitz & Garber, 2003 | NA | NA | ✔ | ✔ | ✔ | **?** | ✔ | ✔ | ✔ | ✔ | ✔ | 88.8 | Low |
| Kasen et al, 2012 | ✔ | ✖ | ✖ | ✔ | ✔ | **?** | ✔ | ✔ | ✔ | ✔ | ✔ | 72.7 | Low |
| Kent, 2020 | NA | NA | **?** | ✔ | ✔ | **?** | ✔ | ✔ | ✖ | ✖ | ✔ | 55.6 | Moderate |
| Kent & Bradshaw, 2020 | NA | NA | **?** | **?** | **?** | **?** | ✔ | ✔ | ✖ | ✖ | ✔ | 33.3 | Moderate |
| Kim, 2002 | NA | NA | **?** | ✔ | ✔ | **?** | ✔ | ✖ | ✔ | ✔ | ✔ | 66.7 | Low |
| Lalayants, 2020 | NA | NA | **?** | ✔ | ✔ | **?** | ✔ | ✔ | ✖ | ✖ | ✔ | 55.6 | Moderate |
| Le, 2007 | NA | NA | **?** | **?** | **?** | **?** | ✔ | ✔ | NA | NA | ✔ | 42.9 | Moderate |
| Liu, 2011 | NA | NA | ✖ | ✔ | ✔ | **?** | ✔ | ✖ | ✔ | ✔ | ✔ | 66.7 | Low |
| Malooly, 2017 | NA | NA | ✔ | ✔ | ✔ | **?** | ✔ | ✔ | ✔ | ✔ | ✔ | 88.9 | Low |
| Miller, 2002 | ✖ | ✔ | ✖ | ✔ | ✔ | ✖ | ✔ | ✔ | **?** | ✖ | ✔ | 54.5 | Moderate |
| Paunesku, 2008 | NA | NA | **?** | ✔ | ✔ | **?** | ✔ | ✔ | ✖ | **?** | ✔ | 55.6 | Moderate |
| Perez, 2009 | NA | NA | ✔ | ✔ | ✔ | **?** | ✔ | ✔ | ✖ | ✔ | ✔ | 77.8 | Low |
| Peterman, 2014 | NA | NA | **?** | ✔ | ✔ | **?** | ✔ | ✔ | **?** | ✔ | ✔ | 66.7 | Low |
| Petts, 2008 | NA | NA | **?** | ✔ | ✔ | **?** | ✔ | ✔ | ✔ | ✔ | ✔ | 77.8 | Low |
| Possel, 2011 | NA | NA | ✔ | ✔ | ✔ | **?** | ✔ | ✖ | **?** | **?** | ✔ | 55.6 | Moderate |
| Ramos-Olazagasti, 2013 | NA | NA | **?** | ✔ | ✔ | **?** | ✔ | ✔ | ✔ | ✔ | ✔ | 77.8 | Low |
| Rasic, 2013 | NA | NA | **?** | ✔ | ✔ | **?** | ✔ | ✔ | ✖ | ✔ | ✔ | 66.7 | Low |
| Reynolds, 2014 | NA | NA | ✔ | ✔ | ✔ | **?** | ✔ | ✖ | ✔ | **?** | ✔ | 66.7 | Low |
| Riley 2016 | NA | NA | ✖ | ✔ | ✔ | **?** | ✔ | ✖ | ✔ | **?** | ✔ | 55.6 | Moderate |
| Sallquist, 2010 | NA | NA | ✖ | **?** | **?** | **?** | ✔ | ✔ | ✔ | ✔ | ✔ | 55.6 | Moderate |
| Smokowski, 2014 | NA | NA | **?** | ✔ | ✔ | **?** | ✔ | ✔ | ✖ | ✖ | ✔ | 55.6 | Moderate |
| Smokowski, 2016 | NA | NA | **?** | ✔ | ✔ | **?** | ✔ | ✔ | ✖ | ✖ | ✔ | 55.6 | Moderate |
| Upenieks, 2021 | NA | NA | ✖ | ✔ | ✔ | **?** | ✖ | ✔ | ✖ | ✔ | ✔ | 55.6 | Moderate |
| van der Jagt-Jelsma, 2017 | NA | NA | **?** | ✔ | ✔ | ✖ | ✔ | ✔ | ✖ | ✖ | ✔ | 55.6 | Moderate |
| van Voorhees, 2008 | NA | NA | **?** | ✔ | ✔ | ✔ | ✔ | ✔ | ✖ | ✔ | ✔ | 77.8 | Low |
| Wortman, 2012 | NA | NA | **?** | ✖ | ✖ | ✖ | ✔ | ✖ | ✔ | ✔ | ✔ | 44.4 | Moderate |
| Yang, 2017 | NA | NA | ✖ | ✔ | ✔ | ✖ | ✔ | ✖ | ✔ | ✔ | ✔ | 66.7 | Low |
| Yeterian, 2015 | NA | NA | ✔ | **?** | **?** | ✖ | ✔ | ✔ | ✔ | ✔ | ✔ | 66.7 | Low |

**Notes:** Risk of bias criteria: 1) Two groups similar and recruited from same population, 2) Exposures measured similarly to assign people to exposed and unexposed groups, 3) Exposure measured in valid and reliable way, 4) Identified confounding factors, 5) Stated strategies to deal with confounding factors, 6) Groups/participants free of outcome at start of study, 7) Outcomes measured in valid and reliable way, 8) Follow-up time reported and sufficiently long enough for outcomes to occur, 9) Follow-up completed and if not, reasons for loss described and explored, 10) Strategies to address incomplete follow-up used, and 11) Appropriate statistical analysis used**;** Answers: No = ✖, Yes = ✔ , Unclear = **?**, Not applicable = NA

**Table 2. Randomized controlled trial studies’ risk of bias criteria and assessment**

| **Studies** | **Risk of bias criteria** | | | | | | | | | | | | | **Risk of bias score (%)** | **Risk of bias rating** |
| --- | --- | --- | --- | --- | --- | --- | --- | --- | --- | --- | --- | --- | --- | --- | --- |
|  | **1** | **2** | **3** | **4** | **5** | **6** | **7** | **8** | **9** | **10** | **11** | **12** | **13** |  |  |
| Armento, 2012 | ✔ | **?** | ✔ | ✖ | ✖ | **?** | **?** | ✔ | ✔ | ✔ | ✔ | ✔ | ✔ | 61.5 | Moderate |
| Charkhabi, 2014 | **?** | **?** | ✔ | ✖ | ✖ | **?** | **?** | **?** | ✔ | **?** | **?** | ✖ | **?** | 15.4 | High |
| Chen, 2005 | **?** | **?** | ✔ | ✖ | ✖ | **?** | **?** | **?** | ✔ | ✔ | **?** | ✔ | **?** | 30.8 | High |
| Chen, 2009 | **?** | **?** | **?** | ✖ | ✖ | **?** | **?** | **?** | **?** | ✔ | **?** | ✔ | **?** | 15.4 | High |
| Chen, 2018 | **?** | **?** | ✔ | ✖ | ✖ | **?** | **?** | **?** | **?** | ✔ | **?** | ✔ | **?** | 23 | High |
| Heidari, 2019 | **?** | **?** | **?** | ✖ | ✖ | **?** | **?** | **?** | **?** | **?** | **?** | ✔ | **?** | 7.7 | High |
| Khaki, 2021 | **?** | **?** | **?** | **?** | **?** | **?** | **?** | **?** | **?** | ✔ | **?** | ✔ | **?** | 15.4 | High |
| Pandya, 2021 | ✔ | ✔ | ✔ | ✖ | ✖ | **?** | ✔ | ✔ | ✔ | ✔ | ✔ | ✔ | **?** | 69.2 | Low |
| Rickhi, 2015 | ✔ | ✔ | ✔ | ✖ | ✖ | ✔ | ✔ | **?** | ✔ | ✔ | ✔ | ✔ | **?** | 84.6 | Low |
| Scott Richards, 2006 | **?** | **?** | **?** | ✖ | ✖ | **?** | **?** | **?** | **?** | ✔ | ✔ | ✔ | ✔ | 30.8 | High |
| Vazifeh Doust, 2020 | **?** | **?** | ✔ | ✖ | ✖ | **?** | **?** | **?** | **?** | **?** | **?** | **?** | **?** | 7.7 | High |
| Wachholtz, 2005 | **?** | **?** | **?** | ✖ | ✖ | **?** | ✔ | **?** | ✔ | ✔ | **?** | ✔ | ✔ | 38 | High |
| Wachholtz, 2008 | ✔ | **?** | ✔ | ✖ | ✖ | **?** | ✔ | **?** | ✔ | ✔ | ✔ | ✔ | ✔ | 61.5 | Moderate |

**Notes:** Risk of bias criteria: 1) Randomized intervention assignment, 2) Concealed treatment group allocation, 3) Treatment groups similar at baseline, 4) Blind to treatment assignment, 5) Those delivering treatment blind to treatment assignment, 6) Outcomes assessors blind to treatment assignment, 7) Treatment groups treated identically, 8) Follow-up completed and if not, between group follow up differences described and analysed, 9) Participants analyzed per group randomisation, 10) Outcomes measures same across groups, 11) Outcomes measured in reliable way, 12) Appropriate statistical analyses used, 13) Appropriate trial design or deviations accounted for; Answers: No = ✖, Yes = ✔ , Unclear = **?**

**Table 3. Pre-post and quasi-experimental studies’ risk of bias and assessment**

|  | **Risk of bias criteria** | | | | | | | | | **Risk of bias score (%)** | **Risk of bias rating** |
| --- | --- | --- | --- | --- | --- | --- | --- | --- | --- | --- | --- |
| **Study** | **1** | **2** | **3** | **4** | **5** | **6** | **7** | **8** | **9** |  |  |
| Anastasi, 2008 | ✔ | **?** | **?** | ✔ | **✖** | **?** | ✔ | **?** | **?** | 33.3 | Moderate |
| Dami, 2019 | ✔ | **?** | **?** | ✔ | ✔ | **?** | ✔ | **?** | ✔ | 55.6 | Moderate |
| Ebrahimi, 2015 | ✔ | **?** | **?** | ✔ | ✔ | **?** | **?** | ✔ | **?** | 44.4 | Moderate |
| Hajra, 2021 | ✔ | NA | NA | **✖** | ✔ | NA | NA | ✔ | **?** | 60 | Moderate |
| Kadafi, 2021 | **?** | **?** | **?** | ✔ | ✔ | **?** | ✔ | **?** | ✔ | 44.4 | Moderate |
| Khubalkar, 2009 | ✔ | NA | NA | **✖** | ✔ | NA | NA | **?** | **?** | 40 | Moderate |
| Klawonn, 2019 | ✔ | NA | NA | **✖** | ✔ | NA | NA | **?** | **?** | 40 | Moderate |
| Lolla, 2018 | ✔ | NA | NA | **?** | ✔ | NA | NA | **?** | **?** | 40 | Moderate |
| Maddix, 2018 | ✔ | **?** | **?** | ✔ | ✔ | **?** | ✔ | **?** | ✔ | 55.6 | Moderate |
| Mastropieri, 2015 | ✔ | NA | NA | **✖** | ✔ | NA | NA | ✔ | ✔ | 80 | Low |
| Penberthy, 2017 | ✔ | NA | NA | **✖** | ✔ | NA | NA | ✔ | ✔ | 80 | Low |
| Safara 2012 | ✔ | **?** | **?** | ✔ | ✔ | **?** | **?** | **?** | **?** | 33.3 | Moderate |
| Singh, 2020 (1) | ✔ | NA | NA | **✖** | ✔ | NA | NA | ✔ | ✔ | 80 | Low |
| Singh, 2020 (2) | ✔ | NA | NA | **✖** | ✔ | NA | NA | ✔ | ✔ | 80 | Low |
| Singh, 2020 (3) | ✔ | NA | NA | **✖** | ✔ | NA | NA | ✔ | **?** | 60 | Moderate |
| Smith, 2011 | ✔ | **?** | **?** | ✔ | ✔ | **?** | ✔ | ✔ | ✔ | 66.7 | Low |

**Notes:** Risk of bias criteria: 1) ‘Cause’ and ‘effect’ varables identified clearly, 2) Participants in comparisons similar, 3) Participants in comparisons received similar treatment/care, 4) Included control group, 5) Multiple pre-post outcomes measures, 6) Follow-up completed and if not, between group follow up differences described and analysed, 7) Outcomes measures same across groups, 8) Outcomes measured in reliable way, and 9) Appropriate statistical analyses used; Answers: No = ✖, Yes = ✔ , Unclear = **?**, Not applicable = NA

## Appendix 4. Gender differences in associations between spirituality and religiosity with depression and anxiety

| Author, year, country | Mental health outcome | Religious and spiritual practices  F M | | Religious and spiritual salience  F M | | | Spiritual wellbeing  F M | | Religious coping (RC)  F M | |
| --- | --- | --- | --- | --- | --- | --- | --- | --- | --- | --- |
| Chan, 2015  USA | Depression  Anxiety | - | - | - | | NE | NA  NA | NA  NA | NA  NA | NA  NA |
|  |  | NA | NA | NA | | NA |  |  |  |  |
| Davis, 2016  USA | Depression  Anxiety | NE | NE | - | | NE | NA  NA | NA  NA | NA  NA | NA  NA |
|  |  | NA | NA | NA | | NA |  |  |  |  |
|  |  |  |  |  | |  |  |  | NA | NA |
| Goeke-Morey, 2014  Northern Ireland | Depression  Anxiety | NA  NA | NA  NA | Fewer symptoms as c/t M)* | | NE* | NA  NA | NA  NA | NA  NA | NA  NA |
|  |  |  |  | Fewer symptoms as c/t M)* | | NE* |  |  |  |  |
| Kent, 2020  USA | Depression  Anxiety | - | NE | NE | | - | NA  NA | NA  NA | NA  NA | NA  NA |
|  |  | NA | NA | NA | NA | NA |  |  |  |  |
| Le, 2007  USA | Depression  Anxiety | + | - | + | | - | NA  NA | NA  NA | NA  NA |  |
|  |  | NA | NA | NA | | NA |  |  |  |  |
| Malooly, 2017  USA | Depression  Anxiety | NA  NA | NA  NA | NA  NA | | NA  NA | NA  NA | NA  NA | NE | NE |
|  |  |  |  |  |  |  |  |  | NA | NA |
| Miller, 2002  USA | Depression  Anxiety | - | - | +(childhood dep) | -(no childhood dep) | -(childhood dep) | NA  NA | NA  NA | NA  NA | NA  NA |
|  |  | NA | NA | NA | |  |  |  |  |  |
| Perez, 2009  USA | Depression  Anxiety | NE | NE | - | | NE | NA  NA | NA  NA | NA  NA | NA  NA |
|  |  | NA | NA | NA | | NA |  |  |  |  |
| Peterman, 2014  USA | Depression  Anxiety | NA | NA | NA | | NA | NA  NA | NA  NA | NA  NA | NA  NA |
|  |  | + | + | NE | | NE |  |  |  |  |
| Petts, 2008  USA | Depression  Anxiety | NE | NE | + (engaged in sexual activity) | | NE | NA  NA | NA  NA | NA  NA | NA  NA |
|  |  | NA | NA | NA | | NA |  |  |  |  |
| Rasic 2013  UA | Depression  Anxiety | - | -( dep), -(reverse, dep) | NE | | NE | NA  NA | NA  NA | NA  NA | NA  NA |
|  |  | NA | NA | NA | | NA |  |  |  |  |
| Smokowski, 2014  USA | Depression  Anxiety | NE*  NE* | NE*  NE* | NA  NA | | NA  NA | NA  NA | NA  NA | NA  NA | NA  NA |

Dep- in participants with depression NA not available. NE no statistically significant effect F female, M male, * depressive and anxiety symptoms measured as emotional and behavioural problems or internalizing symptoms, reverse- presence/absence of depression/ anxiety influencing religious/spiritual domain

## Appendix 5. Race, ethnicity and faith differences in association of spirituality and religiosity with depression and anxiety

| Author, year, country | Mental health outcome | Religious and spiritual practices | | Religious and spiritual salience | Spiritual wellbeing | Religious coping (RC) |
| --- | --- | --- | --- | --- | --- | --- |
| Berry, 2013 | Depression  Anxiety | NE Muslims followed by Christians and Jews | | NA | NA | NE Muslims, followed by Jews and Christians |
| Chan, 2015 | Depression  Anxiety | + ethnic minorities | | NA | NA | NA |
|  |  |  | |  |  |  |
| Davis, 2016 | Depression  Anxiety | NA | | NE Asian American | NA | NA |
|  |  |  |  |  |  |  |
| Horowitz, 2003 | Depression  Anxiety | -, -(reverse) Christian faith | | NA | NA | NA |
|  |  | NA | |  |  |  |
| Le, 2007 | Depression  Anxiety | -European Americans | + African Americans | NA | NA | NE race, ethnicity |
|  |  | NA | NA |  |  | NA |
| Malooly, 2017 | Depression  Anxiety | NA | | NA | NA | NA |
| Miller, 2002 | Depression  Anxiety | - Catholic denomination (males with childhood dep) | NE protestant denomination | NA | NA | NA |
|  |  | NA | |  |  |  |
| Peterman, 2014 | Depression  Anxiety | NA | | NA | NA | NA |
|  |  | NE ethnicity or religious denomination | | NE ethnicity or religious denomination |  |  |
| Petts, 2008 | Depression  Anxiety | -White, Black, Latino | + Asian | NA | NA | NA |
|  |  | NA |  |  |  |  |
| Ramos-Olazagasti, 2013 | Depression  Anxiety | +* latino | | NA | NA | NA |
|  |  | +* latino | |  |  |  |
| Salliquist, 2010 | Depression  Anxiety | + (reverse)* Muslim faith | | NA | NA | NA |
|  |  | + (reverse)* Muslim faith | |  |  |  |
| Van der Jagt-Jelsma, 2017 | Depression  Anxiety | NE* Christian faith | | NE* Christian faith | NA | NA |
|  |  | NE* Christian faith | | NE* Christian faith |  |  |
| Wortman, 2012 | Depression  Anxiety | NA | | NA | NA | + spiritual struggles, Catholic faith |
| Yang, 2017 | Depression  Anxiety | NE, NE (reverse) Buddhism or Daoism | | -, -(reverse) Buddhism or Daoism | NA | NA |
|  |  | NA | | NA |  |  |

NA not available. NE no statistically significant effect F female, M male, * depressive and anxiety symptoms measured as emotional and behavioural problems or internalizing symptoms, reverse- presence/absence of depression/ anxiety influencing religious/spiritual domain

## Appendix 6. Spirituality/ Religiosity as a moderator between risk factor and depression/anxiety

| Author, year, country | Risk Factor | Religiosity/ Spirituality Aspect | Mental health outcome | Finding | |
| --- | --- | --- | --- | --- | --- |
| Ahles, 2016 | Stress | Religious coping | Depression  Anxiety | + (NRC) | |
|  |  |  |  | NA | |
| Ahmed, 2011 | Homelessness | Religious and spiritual practices and salience | Depression  Anxiety | - | |
|  |  |  |  | NA | |
| Benore, 2008 | Asthma | Religious coping | Depression  Anxiety | + (NRC) | |
|  |  |  |  | + (NRC) | |
| Berry, 2011 | Stress | Religious coping | Depression  Anxiety | - | |
|  |  |  |  | NA | |
| Carpenter, 2012 | Stress | Religious coping | Depression  Anxiety | +(NRC) | |
|  |  |  |  | NA | |
| Cotton, 2013 | Asthma | Religious coping | Depression  Anxiety | + (NRC, spiritual struggles) | |
|  |  |  |  | NE | |
| Harker, 2001 | Migration | Religious and spiritual practices and salience | Depression  Anxiety | - | |
|  |  |  |  | NA | |
| Helms, 2015 | Peer victimization | Religious and spiritual practices and salience | Depression  Anxiety | - | |
|  |  |  |  |  | |
| Horowitz, 2003 | Parental depression | Religious and spiritual practices | Depression  Anxiety | -reverse | |
|  |  |  |  | NA | |
| Kasen, 2012 | Parental depression | Religious and spiritual practices and salience | Depression  Anxiety | - | |
|  |  |  |  | NA | |
| Lalayants, 2020 | Child protection services involved youth | Religious and spiritual practices and salience | Depression  Anxiety | NE | |
|  |  |  |  | NA | |
| Liu, 2011 | Stress | Religious coping | Depression  Anxiety | +* | |
|  |  |  |  | +* | |
| Miller, 2002 | Childhood depression | Religious and spiritual practices and salience | Depression  Anxiety | + F | - M |
|  |  |  |  | NA | |
| Ramos-Olazagasti, 2013 | Migration | Religious practices | Depression  Anxiety | +* | |
|  |  |  |  | +* | |
| Reynold, 2014 | Diabetes, Cystic Fibrosis | Religious coping | Depression  Anxiety | + reverse (NRC) | |
|  |  |  |  | NA | |
| Riley, 2016 | Stress in sexual minority | Religious coping | Depression  Anxiety | NE* | |
|  |  |  |  | NE* | |
| Smokowski, 2014 | Ecological transactions | Religious and spiritual practices and salience | Depression  Anxiety | NE* | |
|  |  |  |  | NE* | |
| Smokowski, 2017 | Ecological transactions | Religious and spiritual practices and salience | Depression  Anxiety | NE* | |
|  |  |  |  | NE* | |
| Van der Jagt-Jelsma 2017 | Psychiatric disorder | Religious and spiritual practices and salience | Depression  Anxiety | NE* | |
|  |  |  |  | NE* | |
| Wortman, 2012 | Loss | Religious coping | Depression  Anxiety | + (spiritual struggle) | |
|  |  |  |  | + (spiritual struggle) | |

NA not available. NE no statistically significant effect NRC negative religious coping F female, M male, * depressive and anxiety symptoms measured as emotional and behavioural problems or internalizing symptoms, reverse- presence/absence of depression/ anxiety influencing religious/spiritual domain
